# Resource asynchrony and landscape homogenization as drivers of virulence evolution: The case of a directly transmitted disease in a social host

**DOI:** 10.1002/ece3.11065

**Published:** 2024-02-20

**Authors:** Tobias Kürschner, Cédric Scherer, Viktoriia Radchuk, Niels Blaum, Stephanie Kramer‐Schadt

**Affiliations:** ^1^ Department of Ecological Dynamics Leibniz Institute for Zoo and Wildlife Research Berlin Germany; ^2^ Plant Ecology and Nature Conservation University of Potsdam Potsdam Germany; ^3^ Institute of Ecology Technische Universität Berlin Berlin Germany

**Keywords:** dynamic landscapes, evolution, global change, host–pathogen dynamics, virulence

## Abstract

Throughout the last decades, the emergence of zoonotic diseases and the frequency of disease outbreaks have increased substantially, fuelled by habitat encroachment and vectors overlapping with more hosts due to global change. The virulence of pathogens is one key trait for successful invasion. In order to understand how global change drivers such as habitat homogenization and climate change drive pathogen virulence evolution, we adapted an established individual‐based model of host–pathogen dynamics. Our model simulates a population of social hosts affected by a directly transmitted evolving pathogen in a dynamic landscape. Pathogen virulence evolution results in multiple strains in the model that differ in their transmission capability and lethality. We represent the effects of global change by simulating environmental changes both in time (resource asynchrony) and space (homogenization). We found an increase in pathogenic virulence and a shift in strain dominance with increasing landscape homogenization. Our model further indicated that lower virulence is dominant in fragmented landscapes, although pulses of highly virulent strains emerged under resource asynchrony. While all landscape scenarios favoured co‐occurrence of low‐ and high‐virulent strains, the high‐virulence strains capitalized on the possibility for transmission when host density increased and were likely to become dominant. With asynchrony likely to occur more often due to global change, our model showed that a subsequent evolution towards lower virulence could lead to some diseases becoming endemic in their host populations.

## Graphical abstract

During disease outbreaks, pathogenic virulence is a key trait for successful spread. Global change comes with an increasing frequency of novel zoonotic diseases or disease outbreaks. Here, we use an individual‐based host–pathogen model to study how global change drivers affect virulence in a directly transmitted evolving pathogen in a social host. Our results show an increase in pathogenic virulence as an effect of increasing landscape homogenization.

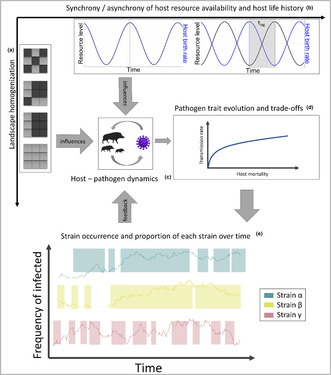

## 1 INTRODUCTION

Global change might exacerbate disease dynamics in the near future, facilitated by land‐use change, habitat encroachment or climate warming (Patz et al., 2004; Wilcox & Gubler, 2005). For example, shifts in phenology, such as advances in the timing of biological events, have been documented extensively (Root et al., 2003). These disturbances will severely influence disease outbreaks via changes in the life history and density, hence availability of hosts. In this context, it is particularly important to understand both, how these disturbances govern the spread and the persistence of pathogens and how they influence the adaptive potential of pathogenic traits, in order to put counteractive measures in place (Griette et al., 2015).

A typical pathogenic trait is its virulence, that is, its ability to negatively affect the fitness of its host (Hudson, 2002). Hence, a key aspect of the invasive success of infectious pathogens in a host population, such as Ebolavirus, SARS‐CoV‐2 or Avian Influenza virus, is balancing the delicate interplay of transmission and host exploitation, also termed the virulence–transmission trade‐off hypothesis (Day, 2003). The virulence–transmission trade‐off hypothesis states that an increase in strain transmission causes shorter infections through higher lethality (Alizon & Michalakis, 2015; Anderson & May, 1982). To persist, a pathogen must find the balance between quick replication and growth in the host, often resulting in severe infections killing its host, while still being able to spread (Visher et al., 2021). This intricate balance can only be kept up by an arms race between hosts' immune reactions and strategies of the pathogen to evade and counteract host resistance, termed adaptive evolution of virulence (Cressler et al., 2016). Consequently, ever‐new pathogenic strains emerge from the wild strain with modulated pathogenic traits, and if the new strain manages to establish, it might have unforeseeable effects on host population and disease dynamics.

Both transmission and virulence are integrally tied to density, spatiotemporal distribution of host individuals, as well as the timing or their life‐history events like birth peaks (Alizon et al., 2009; Cressler et al., 2016), which in return are subject to habitat configuration and spatiotemporal variation in resource availability. Specifically, when the peak of resource availability is synchronized with a biological event, such as seasonal reproduction, this increases host population density and the influx of susceptible individuals, and hence subsequent pathogen transmission (Altizer et al., 2006; van Moorter et al., 2013). On the other hand, drivers such as climate change can shift resource availability and biological events away from each other leading to a mismatch (or asynchrony) between them, which can further decrease host population density or alter host distribution and have subsequent ramifications for pathogen transmission (Duncan et al., 2013; Durant et al., 2007) and virulence evolution (Boots, 2004). Here, theory predicts an evolution towards low virulence through decreased host density and distribution (Boots & Mealor, 2007; Cressler et al., 2016).

**FIGURE 1:**
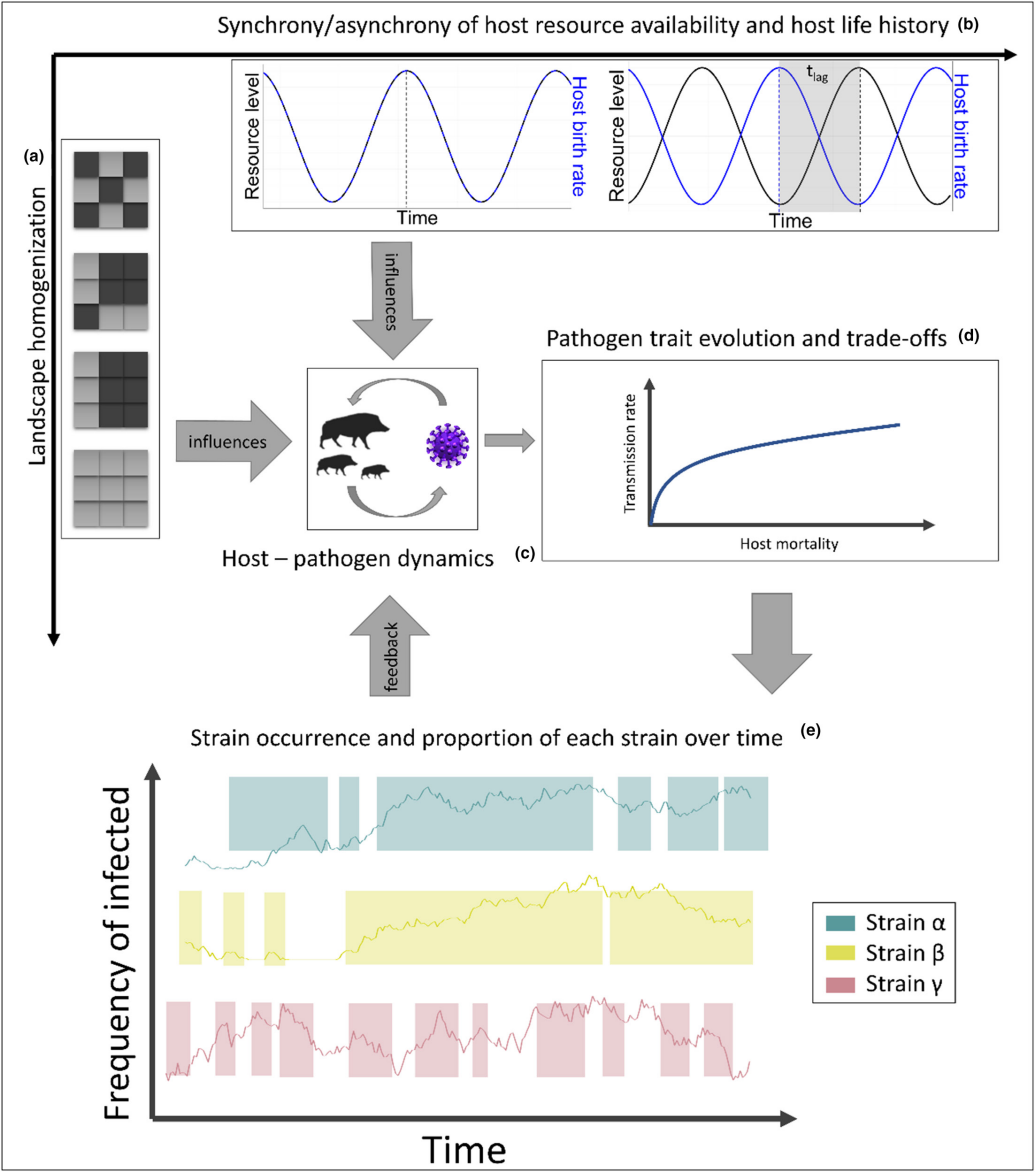
Conceptual figure: Landscape homogenization (a) and synchrony/asynchrony (*t*_lag_) of host life‐history and host resource availability (b) influence host–pathogen dynamics (c) and subsequently the evolution of pathogenic traits (d) that will affect strain occurrence over time where gaps in the background line are times when the strain did not occur in the landscape (e).

The theoretical models of virulence evolution, particularly the classical adaptive dynamics framework, rely on the assumption that mutation of pathogens happens very rarely and that mutations towards new strains can only occur after the dominant strain has reached equilibrium (Dieckmann et al., 2005; Lion, 2018). However, such simplified assumptions are rarely applicable to pathogens in nature, which often are not at equilibrium and undergo transient dynamics, for example, due to temporal and spatial changes in the landscape structure, and therefore, these assumptions are less suited to understand the complex interplay of landscape structure and pathogen dynamics. Due to temporal variation in the landscape, the formation of spatial (Figure 1a) and or temporal (Figure 1b) host hotspots can cascade through the density distribution of potential hosts onto host–pathogen interactions (Figure 1c,d). The formation of hotspots with varying beneficial or detrimental properties for host and pathogen could facilitate the appearance of different pathogenic strains at specific times or locations. The result can be a complex system of different competing and co‐occurring pathogen strains (Figure 1e) with their own spatial and temporal dynamics. The constant emergence, re‐emergence and extinction of pathogenic strains will result in overlap and possible co‐occurrence between different strains, all competing for the same resource (Choua & Bonachela, 2019).

While theoretical studies focus on long‐term predictions of pathogenic strains with evolutionarily stable virulence at equilibrium (Day & Gandon, 2007; Lenski & May, 1994), there is a lack of knowledge linking complex dynamics arising from global change to the evolution of virulence through space and time during an epidemic (Lebarbenchon et al., 2008). Also, links between resources and host density are rarely incorporated into evolutionary models, which typically assume that host density remains at equilibrium. Studies have demonstrated that the virulence of parasites can shift with changes in host densities and resources. Host population dynamics play a crucial role in parasite evolution, including regions of evolutionary bi‐stability, where parasites adapt to their hosts' cycles. This includes phases of high host exploitation, surpassing periods of low virulence (Hite & Cressler, 2018). However, the absence of spatial context and a large scale prompts the question about how the inclusion of spatial effects can contribute to our understanding of host–pathogen interactions. Consequently, there is a need to expand our perspective and investigate these dynamics across various spatial and temporal scales. The examination of how pathogens, hosts and their surroundings interact on a broader scale is imperative for gaining insights into the complexities of epidemics in real‐world conditions.

Here, we go one step beyond the important link between host ecology and parasite evolution by asking how heterogeneously distributed and dynamic resources will impact the evolution of virulence, particularly how temporal mismatches between optimal resource availability and biological events, such as reproduction, affect host–pathogen coexistence and pathogen spread through adaptive virulence dynamics. To this end, we modified an existing spatially explicit individual‐based host–pathogen model of classical swine fever virus in a social mammal, the wild boar (*Sus scrofa*) (Kramer‐Schadt et al., 2009; Kürschner et al., 2021; Lange, Kramer‐Schadt, Blome, et al. 2012; Lange, Kramer‐Schadt & Thulke, 2012; Scherer et al., 2020), and added evolution in pathogen traits leading to multi‐strain outbreak scenarios. With an analysis and integration of different scales, a more comprehensive understanding of the evolutionary dynamics of virulence in epidemics could be achieved, particularly, since empirical studies on the African swine fever virus in wild boar reported the presence of co‐occurring low and highly virulent strains (Portugal et al., 2015). This approach enhances the ecological validity of models and contributes to more effective strategies for disease prevention and control. In accordance with theory, the model has shown for a static host exploitation rate that pathogen extinction is higher in landscapes with randomly distributed and fluctuating resources, but that the formation of disease hotspots facilitates an epidemic rescue for the pathogen when hosts are mobile (Kürschner et al., 2021).

We here hypothesized that dynamic landscapes induce evolution in pathogenic virulence to facilitate host–pathogen coexistence (H1). In more detail, we expect pathogenic virulence to gradually evolve into a system of different viral strains that will co‐occur and persist within the host population in parallel (prediction 1). We also predict that the frequency of ‘host cycle riding’ pathogenic strain emergence will be higher under environmental uncertainty, hence global change effects might lead to higher pathogenic strain emergence (prediction 2), that is, with a higher chance for spillover events.

We further hypothesize that due to the destabilization of the host population under asynchronous dynamics, virulence will evolve to lower levels than under homogeneous and stable resource availability (H2). We expect increasing landscape homogenization and related continuous host contacts to facilitate evolution towards higher pathogenic virulence by increasing the availability of hosts for highly virulent strains (prediction 3), with few dominant strains governing the dynamics (prediction 4).

## 2 METHODS

### 2.1 Model overview

We modified a spatially explicit individual‐based, eco‐epidemiological model developed by Kürschner et al. (2021). It is based on earlier models considering direct transmission between group members and members of any of the eight neighbouring groups that were developed by Kramer‐Schadt et al. (2009), Lange, Kramer‐Schadt, Blome, et al. (2012), Lange, Kramer‐Schadt & Thulke (2012) and Scherer et al. (2020) and includes spatiotemporal landscape dynamics representing changing resource availability, coupled with resource‐based mortality. The model has been parameterized using wild boar demographic rates and CSF infection parameters and can show how individual‐level effects alter disease dynamics on the population and landscape scale. We incorporated evolution of viral traits such as virulence and corresponding trade‐offs with viral transmission (see below). A complete and detailed model description following the ODD (overview, design concepts and detail) protocol (Grimm et al., 2006, 2010) is provided in the Appendix S1 and the model (implementation in NetLogo; Wilensky, 1999) in the Zenodo Database (https://zenodo.org/doi/10.5281/zenodo.10666864).

The model comprises three main components, a host model that is affected by the underlying landscape, an epidemiological pathogen model and a pathogen evolutionary model. Host individuals are characterized by sex, age, location, demographic status (residential and dispersing) and epidemiological status (susceptible, infected and immune). The epidemiological status of the individuals is defined by a SIR epidemiological classification (susceptible, infected and recovered; Kermack & McKendrick, 1927). The pathogen is characterized by strain type, virulence and transmission. The pathogen model alters host survival rates and infection length depending on the pathogen's virulence, while the dynamic landscape features determine host reproductive success. The maximum longevity of any individual host is capped at 11 years; however, given the survival rates based on field data, rarely any individuals reach this age. We record strain occurrences as the number of infected individuals carrying a specific strain and pathogen persistence, measured at the level of simulation runs (see below).

### 2.2 Pathogen dynamics

We determined the course of the disease by an age‐specific case fatality rate and a strain‐specific infectious period. Highly virulent strains are characterized by a short infectious period and low virulent strains by a long infectious period. Transiently infected hosts shed the pathogen for 1 week and gain lifelong immunity (Dahle & Liess, 1992). Infection dynamics emerge from multiple processes: within‐group transmission and individual age‐dependent courses of infection. Within groups, the density‐dependent infection pressure (i.e. the chance of a host individual becoming infected) is determined by a transmission chance and the number of infectious group members carrying the same strain. In this model, we included the dependence of the transmission chance on the strain's virulence, so that the strains with higher virulence have higher transmission chance. Furthermore, we modified the density dependence of the infection pressure to be strain specific to accommodate a lower per‐strain infection density for the following reason: The original model based on a single pathogen strain used the density of infected individuals in a group to infer the likelihood for a susceptible host in that group to become infected based on a binomial model. Our model allows the evolution into 12 (arbitrarily categorized) mutually exclusive different viral strains that are combined into 6 distinct strains, each of the 6 strains is the sum of 2 simulated strains (for more details, see ODD in the Appendix S1). In case of transient infections, if an individual becomes immune, it is also immune to all other strains of the pathogen. The infection pressure, that is, the probability of pathogen transmission to a susceptible host individual, is determined for each strain individually. Differences in strain transmissibility are added to the strain‐specific infection pressure (for details, see ODD in the Appendix S1).

The strain virulence translates directly into infection length, that is, host survival time for individuals that do not recover, where a high virulence results in shorter survival times for the host compared to low virulence. Consequently, the shorter lifetime of a highly virulent pathogen results in a shorter reproductive time span, while making the pathogen highly infective. The overall case fatality of the pathogen (50%), that is, the proportion of hosts that will be lethally infected, is not affected by the virulence of the specific strain.

#### 2.2.1 Evolution of pathogenic traits

Virulence and transmission are emergent properties and are evolving in the model. Each of the six strains has a fixed transmission and survival time value selected from a theoretical trade‐off. Our trade‐off curve is modelled to follow theoretically derived sigmoidal transmission–virulence trade‐off curves usually deployed in virulence evolution models (Alizon et al., 2009; Gandon, 2004) and is applied for each infected host individually through an arbitrarily selected change in the pathogen transmission and survival values (derived from the original values that were parameterized by Scherer et al., 2020) to fit the relationship of per strain transmission and host survival time to the trade‐off curve. During a transmission event, a strain can, with a mutation rate of 0.01, mutate into a new strain with a different virulence. The virulence of the new strain is selected from a normal distribution with a standard deviation *σ* = 1 around the virulence value of the originally transmitted strain and rounded to the closest strain (as integer value), meaning that the new strain will be closely related to the parental strain. The evolution of strains is capped on both strain 1 (being the strain with the lowest virulence) and strain 12 (highest virulence). The boundaries were chosen since the survival time at strain 1 in combination with very low transmission probability results in a chronic long‐term infection that spreads very slowly, and the survival time of individuals infected with strain 6 corresponds exactly with one time step in the model, that is, 1 week corresponding with infectious time, and therefore, a higher virulent strain is not feasible for this model.

### 2.3 Landscape structure and dynamics

**FIGURE 2:**
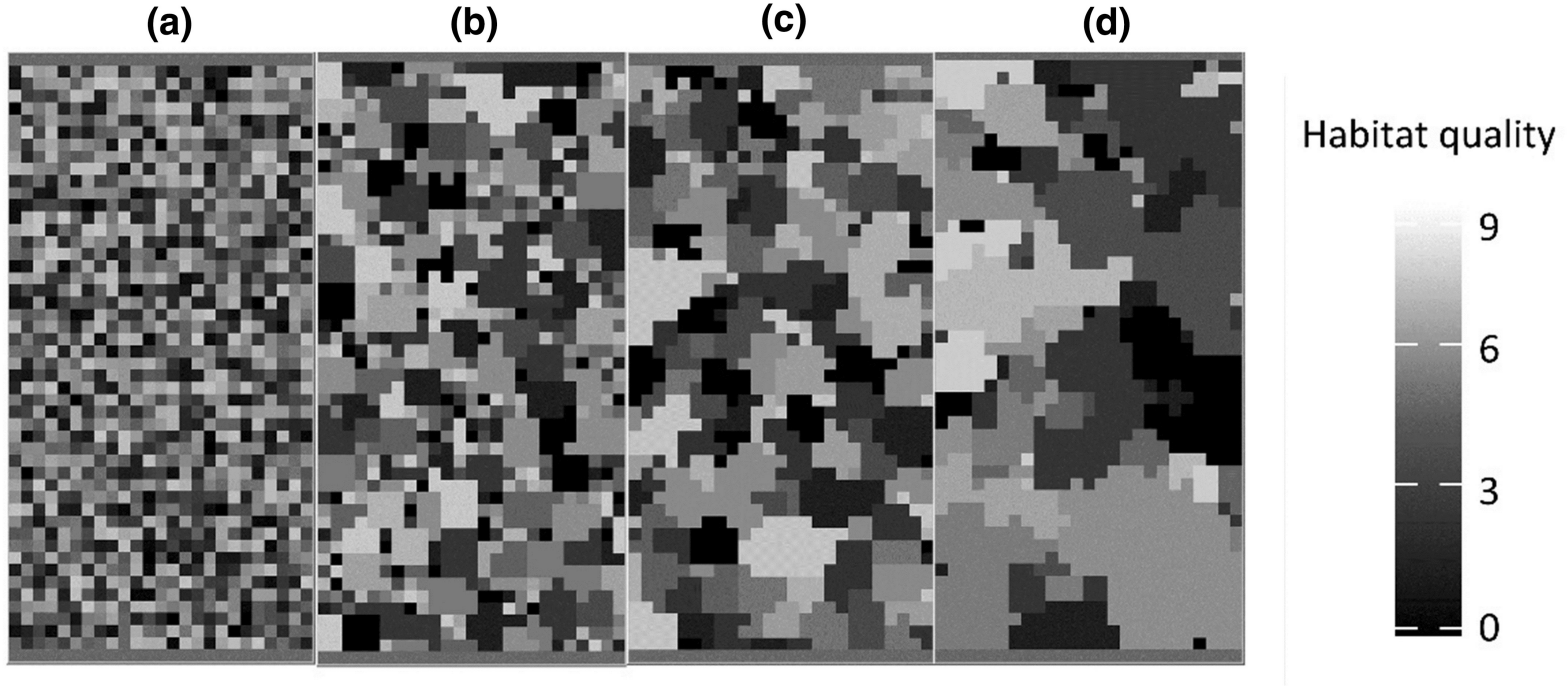
Example landscape configurations and habitat clustering used in the model, with (a) randomly distributed habitat cells, (b) small clusters, (c) medium clusters and (d) large clusters. The colour gradient shows the habitat quality (i.e. the number of breeding females supported by the individual landscape cells). Each cell represents a home range of approx. 4 km^2^ and can host between 1 and 40 group members of different ages and sex classes depending on habitat quality.

The tested landscapes consist of a spatial grid of 1.250 2 km × 2 km cells, each representing the average home range of a social host, for example, a wild boar group (Kramer‐Schadt et al., 2009), totalling a 100 km × 50 km landscape. The landscapes are self‐contained systems without any outside interaction and hard borders. Each cell is characterized by a temporally variable resource availability that represents host breeding capacity and translates directly into host group size, with the minimum being one breeding female per group to a maximum of 9. Resource availability was adapted to achieve the average wild boar density of 5 breeding females per km^2^ (Howells & Edwards‐Jones, 1997; Melis et al., 2006; Sodeikat & Pohlmeyer, 2003). We investigated landscape scenarios with small, medium and large clusters of resource availability as well as a randomly generated landscape (Figure 2), generated in R (R Core Team, 2020) using the NLMR package (Sciaini et al., 2018). To exclude any biases that could stem from different host densities, the mean female breeding capacity (determining the theoretical maximum number of individuals across the landscape) was kept constant at 5 females per km^2^ across the different landscape types (Figure B1 in Appendix S1). The temporal landscape dynamics that were designed to mimic seasonal changes in resource availability by gradually increasing and decreasing resource availability were kept unchanged from the previous model implementation by Kürschner et al. (2021). While the initial landscape creation and configuration were performed with the same mean breading capacity, during the course of the simulation the actual density and distribution of individuals could vary dynamically.

### 2.4 Process overview and scheduling

The temporal resolution of the model equals the approximate pathogen incubation time of 1 week (Artois et al., 2002). The model procedures were scheduled for each step in the following order: pathogen transmission, pathogen evolution, natal host group split of subadult males and females, resource‐based host dispersal, host reproduction, baseline host mortality, strain‐based host mortality, resource‐based host mortality, host ageing and landscape dynamics. Natal group split of males and females was limited to week 17 and week 29 of each year, respectively, representing the observed dispersal period for each sex. Pathogen evolution is not bound to any temporal constraints and happens during transmission events.

#### 2.4.1 Host mortality

Mortality in response to resource availability remained unchanged from the previous model implementation (for details, see ODD in the Appendix S1). Additionally, we added a fixed, strain‐specific mortality that affects the host individuals.

#### 2.4.2 Landscape dynamics with temporal lag

We modelled two levels of asynchrony between reproduction and peak of resource availability, reflected by temporal lag (*t*_lag_, cf. Kürschner et al., 2021): 0% (i.e. full synchrony) and 100% (full asynchrony, meaning lowest resource availability at the time of peak reproduction). The extreme values were chosen because previous studies investigating temporal lag did not show strong effects of the intermediary lags (Kürschner et al., 2021).

### 2.5 Model analysis

Each simulation was run for 100 years, with the virus released in a randomly taken week of the second year (weeks 53–104), to allow the population to stabilize after initialization. The virus was introduced to a set of multiple predefined grid cells resembling wild boar home ranges and containing the individuals of a group in the centre of the landscape to ensure an outbreak. The virus was released in a low‐to‐medium virulence variant at strain 3 (Acevedo, Dillemuth, et al., 2019; André & Hochberg, 2005; Hite & Cressler, 2018). We ran 25 repetitions per combination of landscape scenarios (four levels: small clusters, medium clusters, large clusters and random landscape) and asynchrony (two levels: *t*_lag_ 0% and *t*_lag_ 100%). At each time step, we also recorded the strain occurrence (i.e. if a strain was present in any landscape cell) and number of infected hosts per strain. We further calculated the proportional contribution of each strain to the pool of infected hosts by calculating the ratio of the hosts infected with each strain to the total number of hosts infected with all strains, at each time step. To highlight differences in strain composition between fully synchronous and fully asynchronous scenarios, we subtracted the mean strain proportion in asynchronous scenarios from the mean proportion in synchronous scenarios. For more clarity, we categorized all viral strains into three categories: low virulence strains; medium virulence strains; high virulence strains, each compartment summing the outcomes of two of the six strains modelled.

## 3 RESULTS

### 3.1 Categorized infection trends and strain occurrence

**FIGURE 3:**
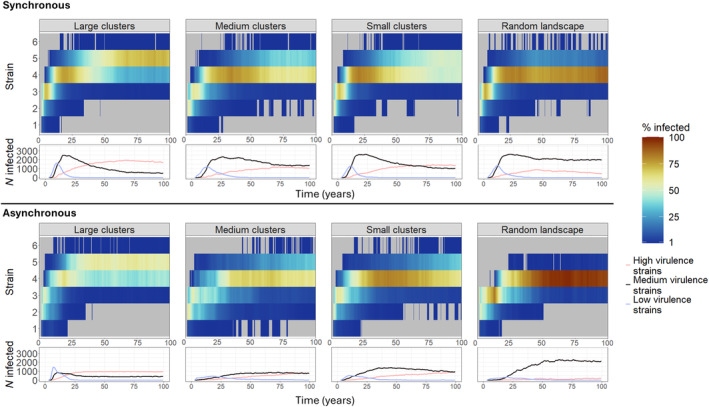
Temporal trends (bottom panels) of the number of hosts infected with the strains of three virulence categories, low (blue), medium (black) and high (red) virulence over time. The top half shows the trends for synchronous host reproduction (*t*_lag_ = 0) and the bottom half for asynchronous host reproduction (*t*_lag_ = 100) scenarios. Occurrence and dominance of the different virulence strains in synchronous (*t*_lag_ = 0, top, top panel) and asynchronous (*t*_lag_ = 100, bottom, top panel) scenarios. Colour gradient represents the proportion of infected individuals of each strain in the landscape. Grey areas represent zero occurrence of the strains.

Our model showed that in synchronous scenarios, highly virulent strains were the least abundant among the three strain categories during the early stages of the epidemic. However, these strains became dominant in the later stages of the epidemic in large clustered landscapes (Figure 3, top, lower panel). With increasing landscape homogenization, medium virulence strains in the later stages of the epidemic were usually dominating along with high virulence strains. Across all landscapes, low virulent strains only occurred in high prevalence in the early stages of the epidemic but reached higher prevalence in less heterogeneous landscapes. Highly virulent strains were found less often and were less dominant in increasingly heterogeneous landscapes combined with synchronous scenarios (Figure 2, top, upper panel). As indicated by the larger proportion of hosts infected with higher virulent strains, overall, in synchronous scenarios, the virulence of occurring strains increased over time and with increasing landscape homogenization.

**FIGURE 4:**
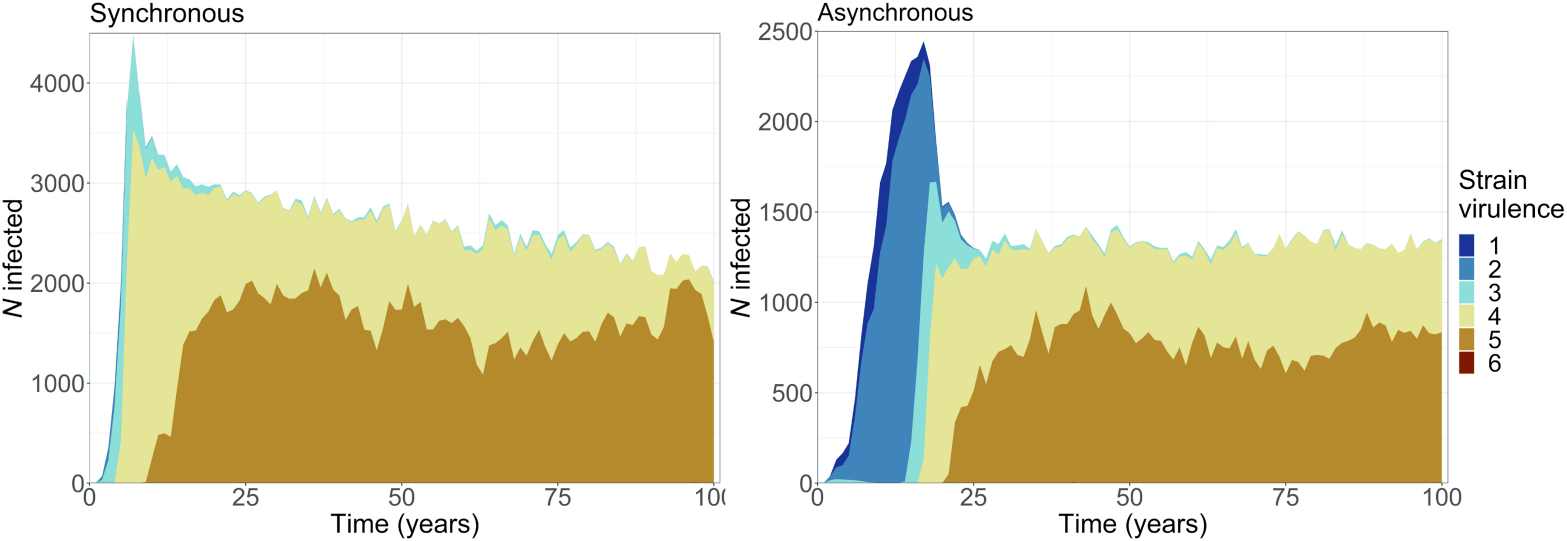
Muller plot for a single example run in a large clustered landscape in synchronous (left) and asynchronous (right) scenarios, showing the number of infected individuals for each strain (colour) over time aggregated as annual mean.

In scenarios with asynchrony, low virulence strains occurred over a longer time period and were more prevalent in the host population, while medium and highly virulent strains increased in prevalence later on (Figure 2, bottom, lower panel). Furthermore, prevalence of all strain categories was lower throughout the simulations when directly compared to the ‘synchronous’ scenarios. A clear shift towards a dominance of highly virulent strains only occurred in the less heterogeneous, large clustered landscapes. In more detail, in asynchronous scenarios, we observed a similar increase in the occurrence of highly virulent strains with landscape homogenization, even though this happened more slowly compared to synchronous scenarios. Furthermore, there was a temporal delay in the strain occurrence within the more homogenous landscape between synchronous and asynchronous scenarios, as can be seen in a direct comparison of the per‐strain infection counts of synchronous and asynchronous scenarios over time (Figure 4) where higher virulent strains occurred later during asynchrony. A general comparison of the proportional strain contribution in asynchronous versus synchronous scenarios further showed that the occurring strains were of lower virulence in asynchronous scenarios in the case of the more heterogeneous landscapes (Figure 2, Figure B3 in Appendix S1).

## 4 DISCUSSION

To extend the understanding of pathogen evolution and spread during epidemics under global change drivers, we implemented virulence evolution in an individual‐based model simulating an interdependent, tri‐trophic system (landscape resources–host–pathogen) utilizing classical swine fever in wild boar as a model system to demonstrate how individual‐level effects can influence the dynamics of disease at both the population and landscape levels. In accordance with our hypotheses, we found an increase in pathogenic virulence and a subsequent shift in strain dominance with increasing landscape homogenization.

Landscape homogenization alters the density distribution of susceptible host individuals by increasing host connectivity, which subsequently can lead to more infection events and viral mutations. Large areas of similar habitat quality that can support numbers of individuals and, therefore, quasi‐homogenous contact rates within these large clusters can facilitate the spread of a pathogen in cases of a large number of individuals (i.e. high habitat quality) as well as forming temporary barriers in cases of low habitat quality (i.e. low number of individuals), hindering the spread of a pathogen. Our results support that high host density and connectivity are the most important factors that affect the emergence of high virulence in directly transmitted diseases under classical transmission–virulence trade‐offs (Castillo‐Chavez & Velasco‐Hernández, 1998). Both drivers of global change jointly affect pathogen virulence: increasingly heterogeneous (e.g. fragmented) landscapes resulted in lower virulence. Similarly, the higher asynchrony between reproduction and resource peak, as is expected to occur more often with climate change (Both et al., 2006), led to lower average strain virulence. Interestingly, under asynchrony, we found a higher proportion of strains co‐occurring in the more homogeneous landscapes (i.e. large clusters) compared to the heterogenous random landscape, indicating that isolated disease hotspots could facilitate the persistence of different viral strains (Kürschner et al., 2021).

The sigmoidal trait relationship that we used for our model (Alizon et al., 2009) suggests that as virulence increases, transmission may initially be quicker due to increased shedding, but would eventually plateau as host mortality limits further spread. Conversely, a highly transmissible but minimally virulent pathogen may face saturation effects, reducing its potential for widespread transmission. It is, however, essential to highlight that alternative trade‐off functions could yield different outcomes (Acevedo, Dillemuth, et al., 2019). For instance, a linear trade‐off might imply a more straightforward relationship, whereas a concave trade‐off could suggest that moderate levels of both virulence and transmission are most advantageous.

As long as host populations in our model are distributed heterogeneously, mean pathogenic virulence remains similar, with little change from completely heterogeneous, that is, random landscapes, to the less heterogeneous medium habitat clusters. However, in large clusters, a clear increase in mean virulence was apparent, showing that there is a threshold in landscape homogeneity not only enhancing pathogen spread but also evolution towards higher virulence. These modelling findings are consistent with previous research on thresholds in pathogen transmission and functional connectivity. For example, homogenous landscapes have been shown to facilitate the spread of rabies virus in raccoons (*Procyon lotor*) (Brunker et al., 2012) or *Mycobacterium tuberculosis* in badgers (*Meles meles*) (Acevedo, Prieto, et al., 2019), while more heterogeneous landscapes have been shown to limit the spread of highly virulent pathogens (Lane‐deGraaf et al., 2013). Host–pathogen interactions – in directly transmitted diseases – occur at distinct points in time and locations, with the spatial and temporal variability in the availability of susceptible hosts being one of the governing factors of a successful transmission (Hudson, 2002; Ostfeld et al., 2005; Real & Biek, 2007). Consequently, homogenous landscapes and their lack of barriers allow more virulent pathogen strains to infect a sufficient number of hosts to persist in those landscapes. On the contrary, in heterogeneous landscapes, small clusters of high host density in a matrix of low density cause the extinction of highly virulent strains. This ‘dilution’ pattern can be explained by the short survival time of individuals in the matrix that form an immunity belt around the clusters and prevent spread between clusters (Marescot et al., 2021). Hence, in parallel with the ‘dilution hypotheses’ at the community scale, heterogeneous or ‘diverse’ landscapes provide less competent hosts for an epidemic (Civitello et al., 2015; Patz et al., 2004) and have also been shown for metapopulation systems (Becker & Hall, 2016; Leach et al., 2016).

Increasing landscape homogenization also resulted in higher mean virulence in scenarios with asynchrony between host life history and resource availability (prediction 3). Even though overall susceptible host density was lower in asynchronous scenarios, the high connectivity in more homogenous landscapes allowed for higher virulent strains to persist at high prevalence. In the more homogenous, but still clustered, landscape, composed of large areas of high habitat suitability, the virulence of occurring strains was similar between the scenarios with and without synchrony. This indicates a strong effect of landscape configuration.

Interestingly, Kürschner et al. (2021) showed that increasing spatial homogeneity of the landscape affected pathogen persistence negatively without pathogen virulence evolution. One reason behind this difference lies in the temporal differentiation of the strains within the landscapes. During the beginning of an outbreak, the pathogen strains with low virulence are able to spread across the landscape into larger habitat clusters due to the long host survival times. However, once the susceptible host density in one of the surrounding areas has become high enough, highly virulent strains that previously only occurred in low prevalence can outcompete the low virulent strains and increase in prevalence. In other words, when host density temporarily increases, the high virulence strains capitalize on the high possibility for transmission and are likely to become dominant (Altizer et al., 2006; Hite & Cressler, 2018). However, although highly virulent strains became more dominant, lower virulent strains continued to persist within the host population. In line with our findings, the co‐occurrence of high and low virulent strains was also shown for rabbit haemorrhagic disease in the United Kingdom (Forrester et al., 2009) as well as influenza A in wild birds (Olsen et al., 2006).

Furthermore, our results show that, independent of landscape heterogeneity, a single, strain of a pathogen is able to evolve into a complex system of multiple co‐occurring strains with varying virulence (prediction 1). However, while multiple strains co‐occurred at any given time throughout all tested scenarios, we demonstrated that some strains likely become dominant (prediction 2). Similarly, a system of co‐occurring low and highly virulent strains was reported by empirical studies of the African swine fever virus in wild boar (Portugal et al., 2015), a pathogen causing severe diseases with huge economic impact (Artois et al., 2002). In this system, the carriers of low virulent strains could remain infectious over long periods of time (de Carvalho Ferreira et al., 2012) increasing the chance of the pathogen transmission and its mutation into higher virulent strains, which could become dominant over time. In our study, the virulence of the dominant strain was intrinsically linked to the degree of landscape homogenization but was also variable in time. Our findings are consistent with theoretical models that showed an increase in pathogenic virulence over time (Osnas et al., 2015). However, while Osnas et al. (2015) assumed a direct trade‐off between virulence and host movement in homogenous landscapes, here we show that different landscape configurations may lead to the same patterns of increasing virulence without the necessity of such a trade‐off.

On the one hand, our results show that with natural landscapes becoming more fragmented and resources becoming more asynchronous due to global change, a shift towards lower virulent pathogens could be expected. Therefore, some diseases may become endemic in their respective host populations. The longer a pathogen is able to persist within its host population, the higher the risk for spontaneous mutations and the possibility of spillovers to other species. On the other hand, global change will lead to increasing homogenization within those fragments (Patz et al., 2004) and has the potential to increase the average pathogenic virulence with possibly catastrophic effects on wildlife communities. A large variance in virulence has been shown among infected host individuals, where the infection can range from severe to asymptomatic. This variation can be the result of a variety of factors, including not only genetic variation or intraspecific host interactions but also environmental conditions (Ebert & Bull, 2003). Furthermore, an increase in virulence is likely to go hand in hand with higher transmission rates in many diseases (Alizon & Michalakis, 2015; Messinger & Ostling, 2009) which will increase the probability of pathogen spillovers even more. However, it is important to note that this dynamic is also dependent on assumed trait relationships. While pathogen spillovers to other wild or domestic animal populations can have profound social or economic effects (Kamo et al., 2007), the possibly detrimental effects on human health cannot be underestimated. The SARS‐CoV‐2 pandemic has clearly highlighted the importance of understanding factors governing the spread of diseases in wildlife populations and how anthropogenic changes may alter those in the future.

## AUTHOR CONTRIBUTIONS

**Tobias Kürschner:** Conceptualization (equal); formal analysis (equal); software (lead); writing – original draft (lead); writing – review and editing (equal). **Cédric Scherer:** Software (supporting); writing – review and editing (equal). **Viktoriia Radchuk:** Formal analysis (equal); writing – review and editing (equal). **Niels Blaum:** Writing – review and editing (equal). **Stephanie Kramer‐Schadt:** Conceptualization (equal); formal analysis (equal); software (equal); writing – review and editing (equal).

## FUNDING INFORMATION

This work is funded by the German Research Foundation (Deutsche Forschungsgemeinschaft DFG‐GRK ‘BioMove’ 2118/1).

## CONFLICT OF INTEREST STATEMENT

The authors declare no conflicts of interest.

## Supporting information


Appendix S1

## Data Availability

The model implementation in NetLogo and analysis scripts in R are available on Zenodo (https://zenodo.org/doi/10.5281/zenodo.10666864).
