# Supplementary material for: Resource asynchrony and landscape homogenization as drivers of virulence evolution: The case of a directly transmitted disease in a social host
Source: Ecol Evol. 2024 Feb 20;14(2):e11065. doi: 10.1002/ece3.11065 (PMC10877554; doi:10.1002/ece3.11065)
Supplement: Supplementary file 1 — Appendix S1 [file ECE3-14-e11065-s001.pdf]

A Model description (ODD protocol)

The classical swine fever (CSF) wild boar model is a combination of a spatially explicit, stochastic, agent-based model for wild boars (*Sus scrofa* L.) and an epidemiological model for the CSF virus. The model was implemented in NetLogo (Wilensky, 1999) and is documented following the ODD protocol (Grimm et al., 2006, 2010, 2020).

The ODD protocol below includes changes in model description across three wild boar / CSF eco-epidemiological models. The first model SwiFCoIBMove (M1 and black text throughout, Scherer et al. 2020) was developed to assess the effects of host movement and landscape structure on disease dynamics, SwiFCoIBM_dynamic (M2 and blue text throughout, Kürschner et al. 2021) was developed to assess the role of a dynamic resource landscape and temporal mismatches on disease dynamics and SwiFCoIBM_Evo (M3 and green text throughout) was developed to investigate the effect of evolving pathogenic virulence. As a note, while in some instance (as seen for example in 1. Purpose) parts of the previous models are replaced, in other instances and submodels (as seen in 4– *Emergence*) M3 is a mixture of old and new versions. As a second note, specific changes to M1 that are in both M2 and M3 are marked with an orange colour in the text.

1 Purpose

[1]M1: The model aims to assess the role of host movement based on individual movement decisions (imposed, i.e. a correlated random walk, or based on external factors such as habitat and conspecifics) and landscape structure in driving on-going disease dynamics as pathogen persistence and patterns of spread. Specifically, the question will be addressed to what degree a high-resolution representation of the interaction between moving vectors and landscape features is required to capture spatiotemporal disease dynamics in a predictive way.

[1]M2: The model aims to assess the role of a dynamic resource landscape and temporal shifts in resources on disease dynamics as pathogen persistence and patterns of spread through individual movement. Specifically, how seasonal resource availability and the decoupling of resource availability and reproduction alter disease dynamics.

M3: The model investigates the effect dynamic resource landscape and temporal shifts on a multi-strain pathogen system, pathogen evolution and pathogen trade-offs. Particularly, how temporal mismatches between optimal resource availability and biological events affect host-pathogen coexistence and pathogen spread.

[1]This ODD describes M3, so that 'Purpose' for M1 and M2 is obsolete. It is kept in the ODD to better understand how the design of the model developed

2 Entities, state variables and scales

The model entities are spatial units, or grid cells, and wild boars. Grid cells are characterized by habitat quality in terms of breeding capacity, i.e. the number of female boars that are allowed to have offspring (Jedrzejewska et al., 1997). Thereby, local host density is in the model, i.e. increasing numbers of fertile females can breed only until breeding capacity is reached. A grid cell represents about 2 km × 2 km, encompassing an average home range area of a boar group (Leaper et al., 1999).

Wild boars are characterized by sex, age in weeks, location, demographic status (e.g. breeding, dispersing and ranging) and health status. Three age classes are distinguished: piglet (< 34 weeks), subadult (between 34 weeks and < 1 year for females and < 2 years for males) and adult. Location is defined by the grid cell the wild boar inhabits. The health status of the individuals is described by an SIR epidemiological classification (susceptible; transiently infected; lethally infected with individual infectious period; immune by surviving the infection or by maternal antibodies). Females, which are at least subadult, may be assigned as breeders according to the breeding capacity of their family group's cell. Subadult wild boars may disperse during the dispersal period dependent on their sex and demographic status (disperser or non-disperser). In the model versions simulating explicit movement, males become ranging individuals (i.e. move solely) once they are adult and thereby change their locations every time step.

M1, M2, M3: One time step corresponds to the approximate CSF incubation time of one week (Artois et al., 2002; Moennig et al., 2003) while simulations usually run for 12 years (624 weeks), 50 years (2600 weeks) or 100 years (5200 weeks) with the virus being released in the second year (week 53-104) to a defined boar group to ensure the same distance to the model borders or the centre of the landscape and an established spatial population structure. The model landscape consists of 100 km × 50 km (50 × 25 grid cells).

3 Process overview and scheduling

[2]M1: Each time step, the following procedures are executed by the wild boars in the given order: pathogen transmission, ranging movement, natal dispersal of males and females, respectively, reproduction, mortality, ageing, and disease course (Figure A1)

In the first week of each year, females are assigned to breed. Natal dispersal of males and females was limited to week 17 and week 29 of the year, respectively.

[2] This ODD describes M3, so that 'Process overview and scheduling' for M1 is obsolete. It is kept in the ODD to better understand how the design of the model developed

M2 & M3: Each time step, the following procedures are executed by the wild boars in the given order: pathogen transmission, ranging movement, natal dispersal of males and females, resource-based host movement, host reproduction, general host mortality, resource-based host mortality, ageing, and disease course (Figure A1.1 (M2) A1.2 (M3)).

M2 & M3: Females are assigned to breed on a week to week basis, with the prerequisite that they have not bread previously in the current year.

4 Design concepts

Basic principles

M1, M2 & M3: Both processes of the host and the pathogen are simulated with a given stochasticity which resembles natural conditions. Disease transmission for groups is modelled density-dependent (see Eq. A1 and A2). Heterogeneous landscape scenarios were simulated using neutral landscape model algorithms.

Emergence

M1, M2 & M3: Wild boar population dynamics emerge from individual behaviour, resulting from age- and sex-dependent movement behaviours (natal dispersal and ranging) as well as age-dependent seasonal reproduction, survival probabilities and is mitigated by the underlying dynamic resource landscape. The epidemic course emerges from different virus transmission probabilities, wild boar movement behaviours (only M1 & M2) as well as individual stochastic disease courses and infectious periods.

Adaptation

M1 & M2: Movement rules either reproduce observed movement behaviours (phenomenological approach, e.g. correlated random walk with a given directional persistence) or are based on decisions in response to spatial structure or conspecifics (mechanistic approach, e.g. the competition-driven movement rule implicitly seeks to increase habitat quality in relation to conspecifics).

M1, M2 & M3: The number of breeding females per cell is determined by (a) the habitat quality of the cell and M1: (b) the number and age of the reproductive females within the cell.

Objectives

M1 & M2: The exact way individuals decide where to move depend on the applied movement rule (see section "Ranging movement"). In general, ranging individuals (i.e. adult males) using

decision-based movement rules move into the neighbouring cell with the highest weight (which is defined by the applied movement rule). Individuals' decisions are not perfect but may be a random direction depending on the directional persistence ρ (probability to move randomly = 1 - ρ, set to 0.7).

M1, M2 & M3: Dispersing individuals search for empty (unoccupied) cells (in case of female dispersers) or join other groups that are below their cells' carrying capacity (in case of male dispersers).

Learning

M1, M2 & M3: There is no learning implemented in our model.

Prediction

M1, M2 & M3: There is no need to predict future environmental conditions.

Sensing

M1, M2 & M3: Individuals moving through the landscape may base their decisions on the underlying landscape structure in terms of habitat quality (or, synonymously, breeding capacity B) or number of conspecifics. Moving individuals can sense all that information from their own cell and surrounding cells but the decision might be random (implicitly due to insufficient information) depending on the assumed directional persistence ρ.

Interaction

M1, M2 & M3: Based on the transmission rates, interactions between individuals might lead to virus transmission from an infected to a susceptible individual. Reproduction is density-and age-dependent with the oldest females giving birth first. Thus, if the number of reproducible females exceeds a cell's breeding capacity, interaction leads to repressed reproduction.

Stochasticity

M1, M2 & M3: Demographic and behavioural parameters are imposed via probability distributions to account for variation in the biological processes. Stochastic individual disease courses and infectious periods are modelled explicitly because variation in the disease outcome between individuals was identified to be essential for virus endemicity without reservoirs (Kramer-Schadt et al., 2009). Movement decisions are based on a random component (with 30% of the decisions being random).

Collectives

M1, M2 & M3: Individuals that are not ranging between home ranges (i.e. all individuals but adult males) form groups within their cell that experience density-dependent transmission probabilities. These groups are formed by reproducible females and their offspring. During natal dispersal, male subadults may join those groups while female subadults may form new groups.

Observation

M1, M2 & M3: To evaluate model outcomes, we measure several properties of the host-virus system at each time step of the simulation. These outputs include number of individuals for each class of age (piglet, subadult, adult), demographic (resident, ranging) and health status (susceptible, infected, recovered) and their combinations. Furthermore, sex ratio, number of new infections (transient and lethal), number of cells with infectious individuals, the last week of infection and the cumulative time (i.e. how many weeks in total) of infected hosts per habitat cell are recorded. From this output, duration of an outbreak, probability of disease persistence (i.e. the virus being present in the system until the end of simulations), outbreak size for different classes, as well as density distribution of infected hosts can be estimated. Furthermore, we recorded the number of infected individuals separately for each strain of the pathogen and the mean landscape-wide virulence of all infected individuals. Due to the small differences between individual strains, we condensed the 12 individual strains into 6 strains by combining adjacent strains (e.g., strain 1 and strain 2 or strain 7 and strain 8).

5 Initialization

M1, M2 & M3: We simulate landscapes with different levels of spatial heterogeneity in habitat quality: homogeneous (only M1 & M3), random, and two or four clustered landscape scenarios using a random cluster algorithm (small, medium and large clusters, respectively; Saura & Martínez-Millán, 2000; Table A1, Figure B1) generated using the R package NLMR (Sciaini et al., 2018), creating a landscape where the host breeding capacity is grouped into clusters of different sizes. In homogeneous landscapes, one boar family group was allocated to each cell with an average breeding capacity B of 4.5 females, resulting in the reported density of approximately 20 boars per cell or 5 boars per km^2 (European Food Safety Authority, 2009; Howells & Edwards-Jones, 1997; Melis et al., 2006; Sodeikat & Pohlmeyer, 2003). In heterogeneous landscapes a breeding capacity B between 0 and 9 was assigned to each of the 1250 grid cells ($B \in \{0;9\}$). In case B is a floating point number, the number of individuals is determined stochastically based on the remainder. Initial age distribution was obtained from the results of a 100-year model run conducted by (Kramer-Schadt et al., 2009); Table A2), the sex ratio was balanced (i.e. probability of 0.5 to be either male or female). Wild boar density reflects long-term average values of densely

populated Central European habitats (European Food Safety Authority, 2009; Howells & Edwards-Jones, 1997; Melis et al., 2006; Sodeikat & Pohlmeyer, 2003). Group size was initialised according to the cells carrying capacity K_i that is 4.5 times its breeding capacity B_i.

6 Input

M1, M2 & M3: The model does not include external input representing environmental conditions changing over time.

7 Submodels

Initial infection (pathogen release)

M1 & M2: The virus is released to the population by infection of one wild boar group (i.e. cell) in middle of the upper row of home ranges (i.e. cell with the coordinates x = 1 and y = 13) to allow for comparison between runs. The release is scheduled in a random week of the second year of the model run.

M3: The virus is released to the population by infection of several wild boar groups (i.e., cells), starting with the centre of the landscape (i.e., cell with the coordinates x = 24 and y = 13) as well as three randomly selected cells in the vicinity with strain 5. The release is scheduled in a random week of the second year of the model run.

Pathogen transmission

[3]M1 & M2: Virus transmission is modelled stochastically. The transmission parameter determines the weekly probability of being infected by an infectious group mate β_w.

In the model without movement (afterwards referred to as the "classical model"), weekly infection pressure λ_i for each susceptible individual in cell i is determined by the probability of being infected by an infectious group mate β_w (within-group transmission probability) and the probability of being infected by an infectious individual in one of the eight neighbouring cells β_e (between-group transmission probability):

$$\lambda_i = 1 - (1 - \beta_w)^{I_i} \cdot (1 - \beta_e)^{\Sigma_i I_j}$$

[A1]

[3]This ODD describes M3, so that '7.2.Pathogen transmission' for M1 and M2 is obsolete. It is kept in the ODD to better understand how the design of the model developed

where I_i is the number of infectious group mates and I_j is the numbers of infectious hosts in the jth adjacent cell. The resulting probability value λ_i provides the parameter of a binomial chance process to decide whether a susceptible animal will be infected.

The transmission parameters β_w (and thereby β_e since it was fixed as one tenth of β_w) was calibrated in order to reproduce the spreading velocity observed in France (Rossi et al., 2010) with the constant parameter value β_w = 0.0208 within and, hence, β_e = 0.00208 between groups (Kramer-Schadt et al., 2009).

In the model version involving explicit movements presented here, infection might be translocated within the host population. Because ranging movements are performed by adult males only, the homogeneous transmission probability λ_i was transformed to account for these sex-and age-dependent transmission probabilities. Therefore, for all individuals but adult males the weekly probability of being infected by infectious group mates (but not by infectious ranging males) is simply β_w since females and their group follow a staying rule with the almost all contacts within the group (Pepin et al., 2016; Spitz & Janeau, 1990). The accumulated probability λ_i for susceptible non-ranging individuals is thus reduced to:

$$\lambda_i = 1 - (1 - \beta_w)^{I_i} \quad [A2]$$

where is the number of infectious group mates (but not infectious ranging males which do not belong to any group). For adult, ranging males, the individual probability of virus transmission during movements β_m (i.e. being infected by an infectious animal in a cell moving through as well as the probability of infecting a susceptible individual while passing the cell) was fitted to account for the resuming transmission probabilities (i.e. the remaining within- and between-group transmission probabilities of the classical model, see Eq. A2). We calibrated one constant transmission probability (i.e. $\beta_w = \beta_m$, afterwards just referred to as simply β) to result in the comparable distributions of the basic reproduction number (R_0, number of secondary infections) as in the classical model. Using a Kolmogorov-Smirnov test, the constant parameter value β = 0.022 gave the best results for different scenarios of infection and movement rules (Scherer et al., 2020). Individual per step transmission probability $\beta_{m,i}$ is related to the number of steps the animal makes. Therefore, the infection probability for cells passed by infected individuals is scaled to the individual movement distance $d_{m,i}$ (see section "Ranging movement"):

$$\beta_{m,i} = \frac{\beta}{d_{m,i}}. \quad [A3]$$

By doing so, infection probability decreases with increasing movement distance which relates to the time an animal is able to spend within each cell and thus to transmit the virus on its way.

CSF shows a variety of disease courses on the individual level (Depner et al., 1997; Liess, 1987). Therefore, in our model the disease course is stochastically specified for each individual. The disease course submodel is described by two parameters: individual case mortality M and the mean infectious period of lethally infected hosts μ. Upon infection the host is stochastically assigned either as lethally infected (with probability *M*) or as transiently infected (1 - *M*). *M* is age-specific (Dahle & Liess 1992): for adults the probability is decreased to $M_a = M^2$ and for piglets increased to $M_p = \sqrt{M}$ while it is unchanged for subadults $M_s = M$. Transiently infected wild boars first pass through an infectious period of one week and subsequently becomes non-infectious and gain life-long immunity (Artois et al. 2002; Moennig et al. 2003; European Food Safety Authority, 2009). The individual infectious period (m_i in weeks) of lethally infected hosts is drawn from an exponential distribution with the mean specified by parameter μ:

$$m_i = 1 + floor(-(\mu - 0.5) \cdot ln(U(0,1))) \qquad [A4]$$

where *U(0,1)* is a uniformly distributed random number between 0 and 1. To avoid unrealistically long infections, m_i was stochastically assigned until $m_i \leq 10 \times \mu$. Lethally infected hosts remain infectious until death. Offspring from immune female breeders gets maternal antibodies and is thus immune for the first eight to twelve weeks after birth (t_{anti}). The number of weeks of immunisation due to maternal antibodies is randomly assigned for those piglets.

M3: In the model, instead of one single viral strain like in M1 and M2, we added 12 arbitrarily categorized viral strains that differ in their transmission potential and the survival time of the host they infect (Table A5). Similar to the previous model version, weekly infection pressure λ_{is} for each susceptible individual in cell i is determined by the probability of being infected by an infectious group mate β_w (within-group transmission probability) and the probability of being infected by an infectious individual in one of the eight neighbouring cells $\frac{\beta_w}{10}$ (between-group transmission probability):

$$\lambda_{is} = 1 - (1 - (\beta_w + T_s))^{I_s} \cdot (1 - \frac{(\beta_w + T_s)}{10})^{\Sigma_i I_{js}}$$

[A5]

where I_i is the number of infectious group mates and I_j is the numbers of infectious hosts in the jth adjacent cell. The resulting probability value λ_{is} provides the parameter of a binomial chance process to decide whether a susceptible animal will be infected. Due to the model now having multiple strains with different infection probabilities, there is now the subscript s added to all relevant terms, indicating that those values are now strain specific. Therefore, we calculate infection pressures for all strains separately and only take individuals with the respective strains into consideration when determining I_s for within group or I_{Js} for between group transmission.

We added a strain specific factor T_s (Table A5) to infection pressure calculation so ensure transmissibility differences of the individual strains along a trade-off (Table A5), while the base transmission probability β_w (0.0208) remained unchanged from model M1 and M2.

CSF shows a variety of disease courses on the individual level (Depner et al., 1997; Liess, 1987). Therefore, in our model the disease course is specified for each individual. The disease course sub model is described by two parameters: individual case mortality M and the infectious period of lethally infected hosts. One week after infection the host is stochastically assigned either as lethally infected (with probability M) or as transiently infected (1 - M) in which case it becomes non-infectious and gains life-long immunity. M is age specific (Dahle & Liess 1992): for adults the probability is decreased to $M_a=M^2$ and for piglets increased to $M_p=\sqrt{M}$ while it is unchanged for subadults M_s=M. The individual infectious period is strain specific and selected from table A5.

Lethally infected hosts remain infectious until death. Offspring from immune female breeders get maternal antibodies and is thus immune for the first eight to twelve weeks after birth (t_{anti}). The number of weeks of immunisation due to maternal antibodies is randomly assigned for those piglets.

Pathogen evolution

During every transmission event, the pathogen can evolve into a different strain with different strain specific variables compared to the parental strain. Once a host individual is becoming infected by a specific strain, an evolution check is applied, whereby with a mutation rate of 0.01 (table A1) the strain can evolve. If the strain evolves, the new strain is selected from a normal distribution with a standard deviation of 1 around the parental strain. The resulting strain will be closely related to the parental strain. If the transmitted strain does not evolve, it is transmitted directly without any changes to a new host. For the analysis the 12 strains were condensed into 6 strains (e.g., strain 1 and 2 or strain 11 and 12) due to small differences between the strains.

Ranging movement

[4]M1 & M2: While adult females move mainly within their family group (*staying strategy*), adult males follow a *ranging strategy* and move solitary (Spitz & Janeau 1990, Figure A2). Ranging distances vary between males with a mean distance of 24 km per week and rare long-distance behaviour with up to 84 km per week (Morelle et al. 2015). For each adult male *i*, an individual movement distance $d_{m,i}$ was drawn from the median of the Weibull distribution given by *b*(1 - ln(1 - U[0,1]))^k* with a scale of *b* = 26 and a shape of *k* = 1.3, resulting in a mean of 12 cells and

[4]This ODD describes M3, so that '7.3.Ranging movement' for M1 and M2 is obsolete. It is kept in the ODD to better understand how the design of the model developed

truncated to the maximum movement distance D_{max} of 42 cells (with a cell being 2 km in diameter). To study effects of inter-cell movement on disease dynamics, we implemented three different movement strategies. In general, ranging individuals move until the individual weekly movement distance is reached or there is no better decision to make and only into cells with a positive habitat quality ($B > 0$). Individuals stop moving if there is no cell available. If there is a tie (i.e. two or more cells with equal attractiveness), one of those cells is chosen randomly.

M1 & M2: *(a) Correlated Random Walk (CRW):* In a CRW, subsequent movement directions are correlated such that highly correlated movement paths are nearly straight (Turchin 1998; Figure A3a). This movement model improves the RW approach by incorporating directional persistence that moving animals very frequently exhibit (Figure A3; Kareiva & Shigesada 1983). The direction of a step is drawn from a wrapped Cauchy distribution with a mean direction equal to the previous direction (Fletcher 2006):

$$\theta_{t,i} = \theta_{t-1,i} + 2 \cdot arctan\left(\frac{1-\rho}{1+\rho} \cdot \pi \cdot U(-0.5{,}0.5)\right) \qquad [A6]$$

Where $\theta_{t,i}$ and $\theta_{t-1,i}$ are the headings of individual *i* of the next or previous step, respectively, ρ is a parameter related to the concentration around the mean direction (i.e. ρ = 1 results in a straight line and ρ = 0 equals a RW), and U(-0.5,0.5) is a uniformly distributed random number between -0.5 and 0.5. In case individuals reach the border of the simulated landscape, the Individual is reflected in a 90° angle followed by reset and recalculation of $\theta_{t,I}$ to avoid aggregation around the landscape borders.

M1 & M2: *(b) Habitat-Dependent Movement (HDM):* A reasonable hypothesis is that a selective animal should choose high quality cells for its home range. The distribution and abundance of resources greatly affect movement patterns of several animal species and has also been reported for wild boars (Morelle et al. 2015). Assuming higher food resources and better shelter in high quality habitat, individuals decide among their neighbouring cells based on the habitat quality (Figure A3b). The parameter ρ here relates to the accuracy of an individual estimating the quality of the focal and neighbouring cells, with the movement decision in ρ-1 cases being purely random.

M1: *(c) Competition-Driven Movement (CDM):* Effective habitat quality might be lower due to competition with conspecifics. Thus, competition-driven movement is biased to cells with higher net habitat quality and was implemented as negative density dependence. Movement weights were calculated as the ratio of number of conspecifics in the focal cell and the carrying capacity (K), i.e. the potential maximum group size of the cell (Figure A3c).

For all movement strategies, ρ was fixed as 0.7, i.e. 30% of the decisions were made randomly.

M3: No ranging movement was applied.

Natal dispersal

M1, M2 & M3: Herd splitting, where subadult individuals may move together to search for or form new groups, is performed in specified weeks of the simulation. The timing of these events is sex-dependent: Subadult females without offspring perform their natal dispersal in the 29th week of the year, while subadult males disperse during the 17th week. In the given week of the year, all herds to split are extracted, matching the conditions of containing at least a specified number of subadults N_{disp} to move (either male or female). For female dispersal, only cells exceeding the breeding capacity B are evaluated. For each of them, a habitat cell not exceeding carrying capacity K (for males) or without any family group (for females) within a Euclidean distance d_{disp} is selected randomly as new cell for the group, excluding the source cell (Figure A2). If there is no cell fulfilling these conditions, subadults stay within their group's cell.

Reproduction

M1, M2 & M3: Females reproduce once a year, depending on their age class. Individual females, which are at least subadult, reproduce depending on the season with a peak in March and no reproduction in winter from October to December (Boitani et al. 1995) (Table A2).

M1, M2 & M3: In the first week of the year, female individuals are checked for their breeding status; female individuals are checked for their breeding status on a weekly basis. All females not exceeding their habitat cells breeding capacity, starting with the oldest individuals, are allowed to breed.

M1: The week of the year to breed is assigned in the first week of each year according to weekly reproduction probabilities, derived from monthly probabilities and the number of weeks in the month (Table A2).

M2 & M3: If an individual was assigned to breed the chance of reproduction at that time is drawn from monthly probabilities and the number of weeks in the month (Table A2).

M1, M2 & M3: Litter size is drawn from a pre-calculated truncated normal distribution (Table A1,3) and reduced to a constant fraction for infected individuals. Litter size of transient shedders and lethally infected hosts is multiplied with the reduction factor α_f.

M1, M2 & M3: Depending on the disease state of the breeding individual, its piglets disease states are adjusted. Susceptible individuals produce susceptible offspring, immune individuals produce immune offspring with maternal antibodies (see section "Pathogen state transition"). Transient shedders and lethally infected individuals yield offspring, each one lethally infected with a given probability of prenatal infection p_{pi}.

Mortality

M1, M2 & M3: Stochastic baseline mortality is age-dependent and adjusted to annual survival estimates found in the literature (Table A1). Per time step we apply the adjusted age-dependent mortality (m_{week}) to the individual:

$$m_{week} = 1 - (s_{year})^{1/52}. \quad [A7]$$

In addition to the stochastic baseline mortality, each individual may die due to reaching a certain maximum age, or due to a lethal infection after a certain infection time span m (see section ”Pathogen transmission”).

M2 & M3: A selected number of individuals K_{over} (see Resource response for the selection) are subject to a variable, resource-based mortality with an increasing chance of mortality dependent on how long the individual is subject to low resource conditions up to a variable time limit S_{max} as well as the number of individuals (g_{act}) in relation to maximum group (g_{sus}) size and age.

$$m_{resource} = \frac{(g_{act} - g_{sus})}{100} * a * t_{over}$$

[A8]

With a being an age factor corresponding to the individuals being a piglet or adult/subadult and t_{over} the time the individual is part of the K_{over} individual pool. As well as a 100% mortality when t_{over} equals S_{max}.

Ageing

M1, M2 & M3: The ageing process iterates over all individuals. For each individual k, age T_k is incremented one week and disease state transitions are performed. Females become subadult and adult at an age of 34 and 52 weeks, respectively, while males enter the subadult and adult age groups at an age of 21 and 104 week, respectively.

Disease course (pathogen state transition)

M1 & M2: Transient shedders convert to immune after a certain latency period t_{latent}. An individual i protected by maternal antibodies turns susceptible if reaching an age T_i of the protection time of t_{anti} (see section “Pathogen transmission”). After disease state transition the age of the infection is incremented by one week if the individual is not susceptible.

M3: An individual i protected by maternal antibodies turns susceptible if reaching an age T_i of the protection time of t_{anti} (see section “Pathogen transmission”). After disease state transition the age of the infection is incremented by one week if the individual is not susceptible.

Landscape dynamic

M2 & M3: The autocorrelated landscape dynamic is superimposed over the different types of heterogeneous starting landscapes. Throughout each simulated year, resource availability in each cell increases in 5-weeks intervals for approximately 25 weeks and then declines in 5-weeks intervals for the next 25 weeks. The resource availability translates directly into the breeding capacity for each cell and for each increase or decrease the value of the breeding capacity is changed by one. During the increase, the breeding capacity cannot exceed the maximum breeding capacity of 9 and cannot fall below 1 during the decrease i.e., the breeding capacity in a cell designated as habitat cannot become 0 during the course of a year. Additionally, barriers and matrix which have an assigned breeding capacity of 0 cannot become habitat i.e., matrix/barrier cells will always have a breeding capacity of 0. The default landscape dynamic is implemented to follow the host individual's monthly reproduction probabilities (see table A2), where the peak reproduction probability matches in time with the peak resource availability. Furthermore, the landscape dynamic can be temporally shifted away from the peak host reproduction probability in 25% increments (0% – match, 25% mismatch, 50% mismatch, 75% mismatch, 100% – full mismatch). In this case, a full mismatch would mean that the resource availability is at its lowest when the host reproduction probability is at its highest.

The random landscape dynamic functions similar to the autocorrelated variant without a temporal shift. Throughout each simulated year, resource availability in each cell changes randomly in 5-weeks intervals for approximately 50 weeks whereby each cell is allocated a random integer between 1 and 9. At the end of each year, the landscape resets to the original starting landscape.

Resource responses

M2 & M3: If the group size exceeds the carrying capacity K in a cell, a dispersal event will be triggered. The likelihood of dispersal follows an age gradient where older females will try to disperse last. For male individuals, a random habitat cell not exceeding the carrying capacity K will be randomly selected within a Euclidean distance d_{disp} as new cell. For female individuals, the randomly selected habitat cell must fulfil the prerequisite of not hosting another family group. However, if an individual currently has offspring it and the offspring will stay at in their current cell. If there is no suitable habitat cell fulfilling these prerequisites within the distance d_{disp} the individuals will stay. Individuals are continuously selected for dispersal until the group size no longer exceeds the carrying capacity K. For a secondary response after the dispersal event (if applicable), a cell specific number of individuals K_{over} that is the difference between carrying capacity K and group size will be selected. The selection of these individuals is age dependant

where the oldest females are selected last. All selected individuals are subjected to an increasing age-dependant mortality (see mortality).

Figures

Flow chart of the SwiFCoIBMove model and its submodels

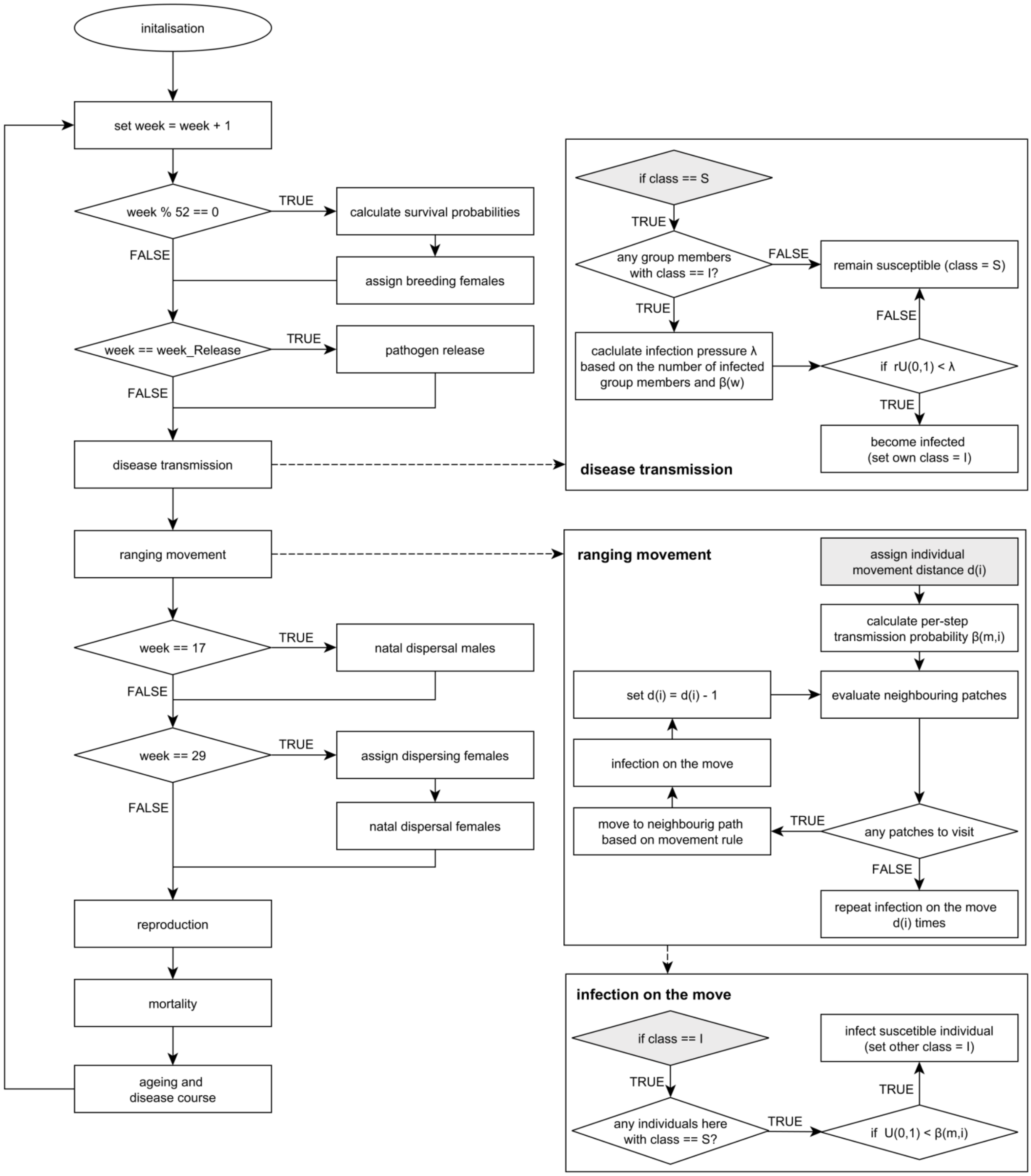

Figure A1: Flow chart of the agent-based, spatially explicit host-pathogen model “SwiFCoIBMove” and its submodels.

Flow chart of the SwiFCoIBM_dynamic model

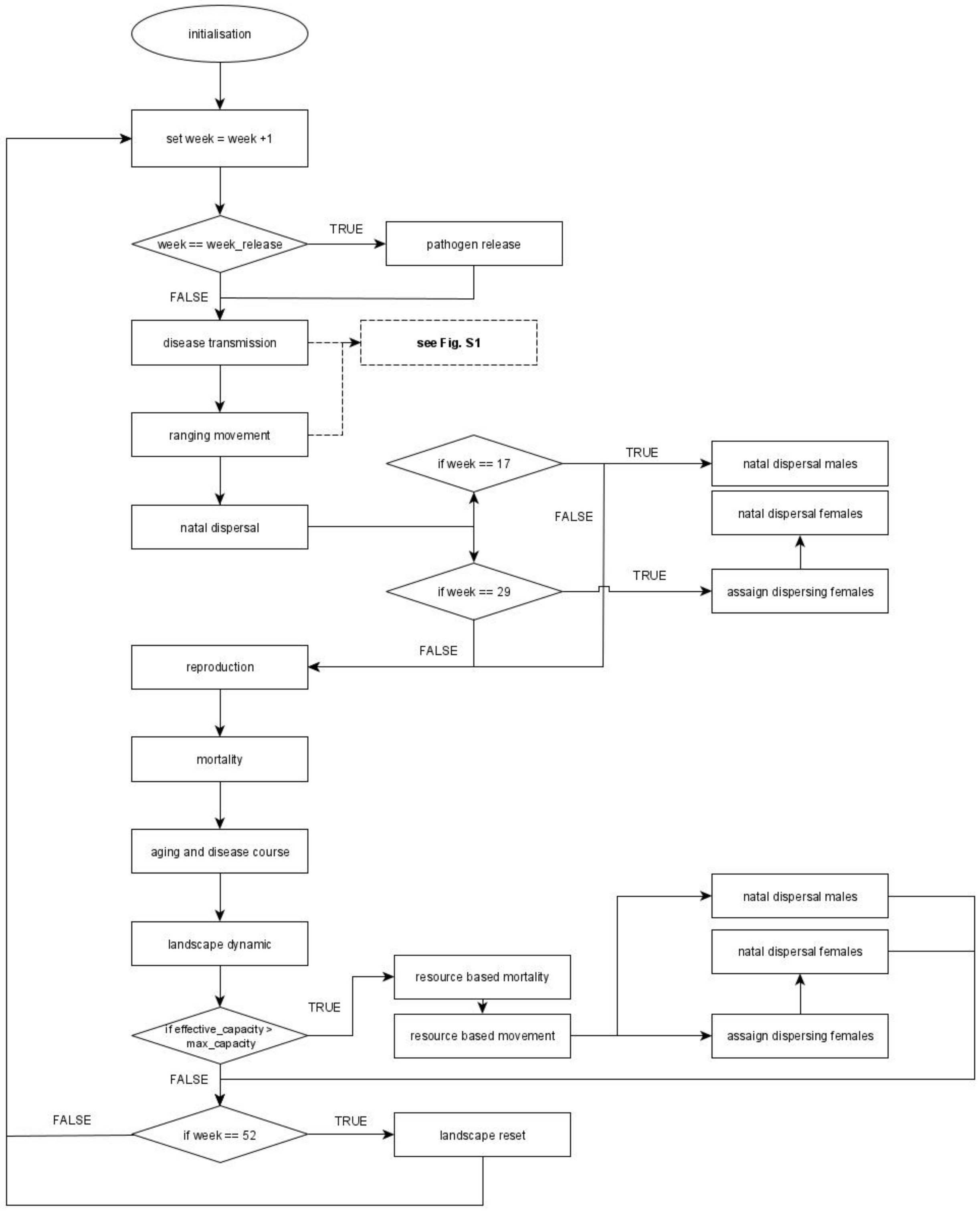

Figure A1.1: Flow chart of the agent-based, spatially explicit host-pathogen model “SwiFCoIB_dynamic”

Flow chart of the SwiFCoIBM_evo model

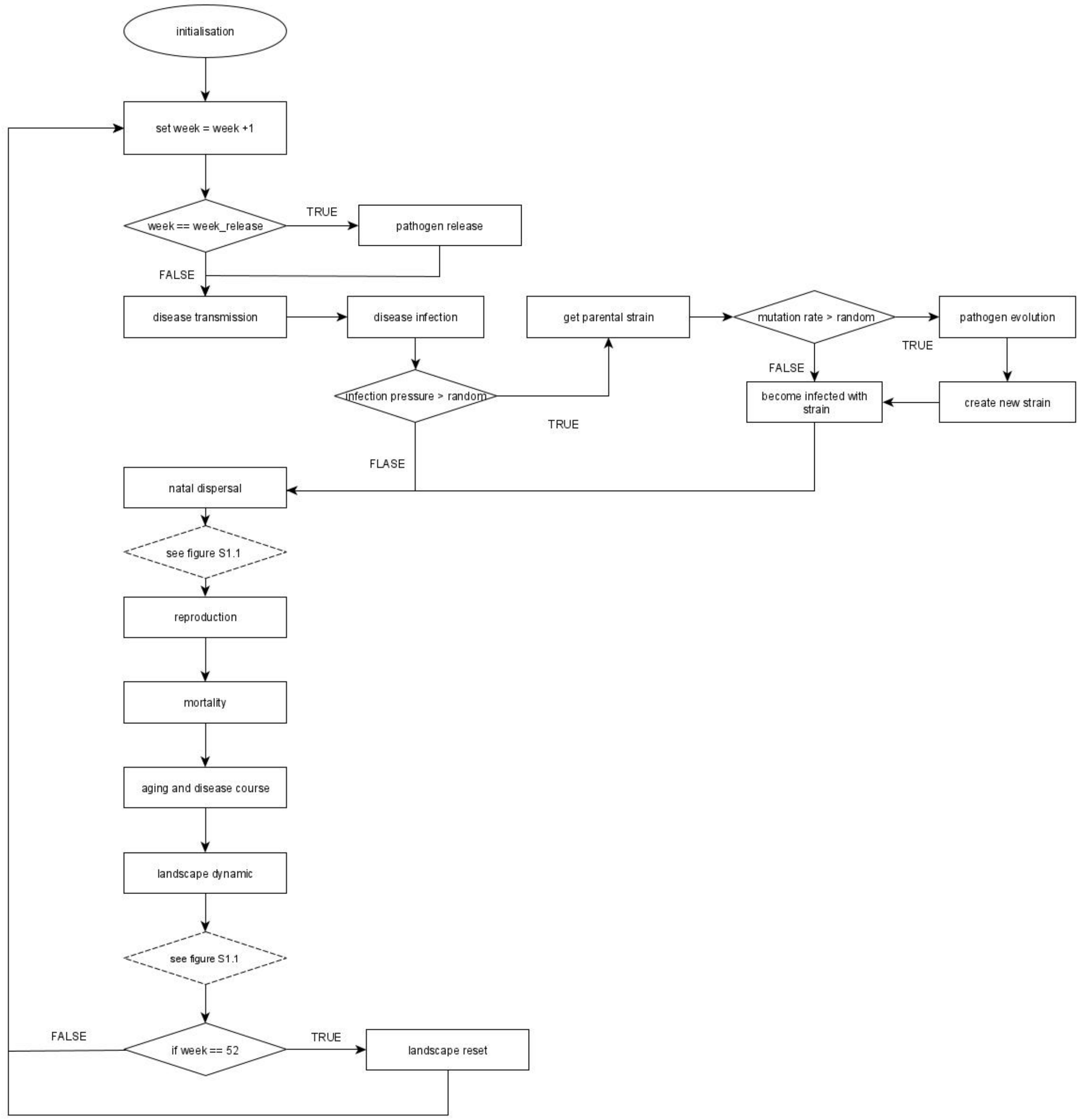

Figure A1.2: Flow chart of the agent-based, spatially explicit host-pathogen model “SwiFCoIB_evo”

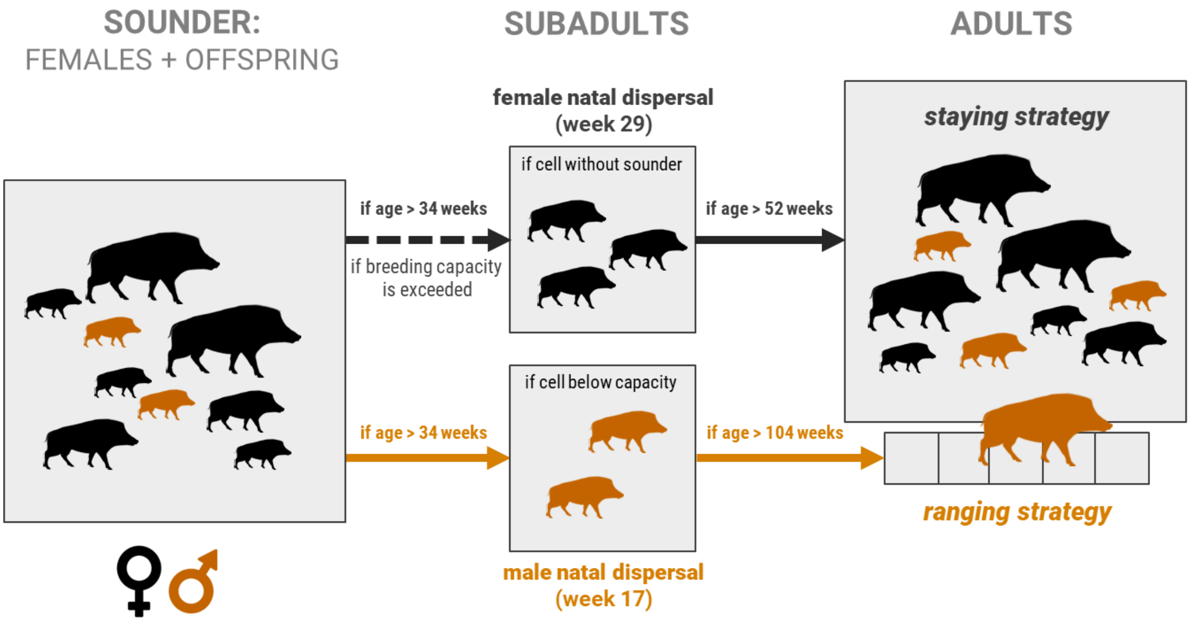

Figure A2: Schematic representation of different natal dispersal events of subadult wild boars and movement strategies of adult wild boars. The group of adult and subadult females and their offspring is called a sounder. If the breeding capacity of the sounders cell is exceeded, subadult females search in groups of 3 or more individuals for an unoccupied cell in a radius of three cells. Female wild boars reach their adulthood earlier (model assumption: if older than 1 year) and follow a staying strategy, thus not moving outside of the sounders cell (grey squares). Male wild boars always separate from the sounder when reaching the subadult age and move in groups of 2 or more to one of the cells below capacity in a radius of 3 cells. When becoming adult, male wild boars move solitary between home ranges.

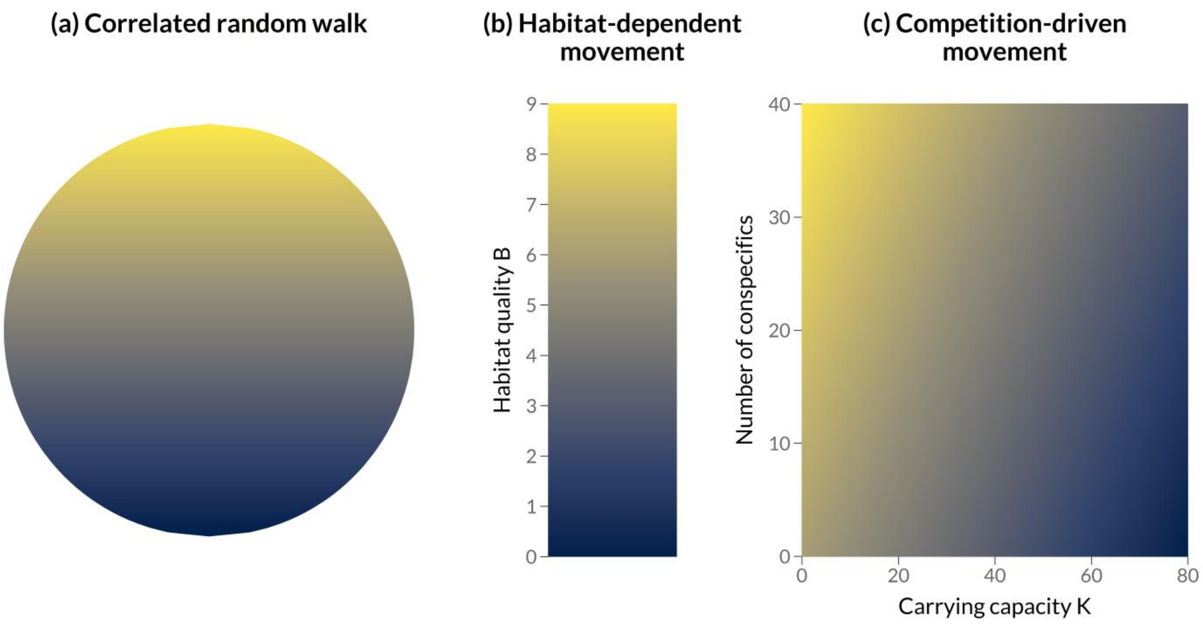

Figure A3: Movement assumptions used to model ranging behaviour of adult males. Probability of movement depends on the **(a)** direction of previous steps (Correlated Random Walk), **(b)** habitat quality *B* or **(c)** carrying capacity *K* in relation to the number of conspecifics. The lighter the colour, the higher the probability the individual chooses this cell. Movement decisions are furthermore driven by a random component determined by ρ.

Table A1: Parameter setup used in the spatially-explicit classical swine fever-wild boar model. M1: Each of the 60 combinations (3 movement strategies × 4 landscape scenarios × 5 case fatality ratios) was repeated 200 times while generating new underlying landscapes depending on the scenario input, resulting in 12.000 runs in total. M2: Each of the 300 combinations (3 movement strategies 3 × landscape dynamic × 4 landscape scenarios × 5 max survival time under low resources × 5 level of resource mismatch) was repeated 25 times, resulting in 22.500 runs in total. M3: Each of the 8 combinations (4 landscape scenarios × 2 landscape dynamic) was repeated 25 times, resulting in 200 runs in total

Parameter	Values	Reference(s)
Longevity	11 years (572 weeks)	(Jezierski, 1977)
Sex ratio	1:1	(e.g. Durio et al., 2014; Fernández-Llario et al., 1999; Moretti, 2014)
Survival probability of adults and subadults	s_{mean}= 0.6; s_{min} = 0.4	(Focardi et al., 1996; Gaillard et al., 1987)
Survival probability of piglets	s_{mean}= 0.5; s_{min} = 0.1	(Focardi et al., 1996)
Reproduction probability	see Table A2	(Boitani et al., 1995)
Breed count distribution	see Table A3	(Bieber & Ruf, 2005)
Movement rule (M1, M2)	CRW (M1,M2)<br>HDM (M1,M2)<br>CDM (M1)	(Fletcher, 2006)
Tendency to move randomly ρ (M1, M2)	0.3	
Mean movement distance D_{mean} (M1, M2)	24 km (12 cells) per week	(Morelle et al., 2015)
Maximum movement distance D_{max} (M1, M2)	84 km (42 cells) per week	(Morelle et al., 2015)
Indiv. weekly ranging distance $D_{m,I}$ (M1, M2)	D_i {1, D_{max}} ~ Wei(26, 1.3)	
Maximal natal dispersal distance d_{disp}	3 cells (6 km)	(Sodeikat & Pohlmeyer, 2003)
Minimum number of dispersers N_{disp}	2 for females; 3 for males	
Case fatality ratio *M*	{0, 0.25, 0.5, 0.75, 1}<br>0.5, 0.5	
Mean infectious period μ (M1, M2)	4 weeks, 6 weeks	
Transmission probability β (M1, M2)	0.022	
Fertility reduction due to infection	0.625	(Kramer-Schadt et al., 2009)
Probability of prenatal infection	0.5	(Kramer-Schadt et al., 2009)
Transient period t_{trans}	1 week	(Artois et al., 2002; Moennig et al., 2003)
Period of maternal antibodies t_{anti}	12 weeks	(Depner et al., 2000)
Simulated years	12 (624 weeks), 50 (260 weeks), 100 (5200 weeks)	

*Continued on the next page

Table A1: Parameter setup – continuation

Parameter	Values	Reference(s)
Pathogen release	random week of the 2nd year	
Mean number of reproductive females	4.5	(European Food Safety Authority, 2009; Howells & Edwards-Jones, 1997; Sodeikat & Pohlmeyer, 2003)
Habitat quality range	[0, 9]	
Initial mean density	5 individuals/km^2	(Kramer-Schadt et al., 2009)
Initial age distribution	see Table A4	(Kramer-Schadt et al., 2009)
Age blur in initial individuals	± 3 weeks	
Landscape dynamic (M2, M3)	Autocorrelated, Random<br>No dynamic	
Max survival time under low resources S_{max}	{5,10,15,20,25} weeks<br>10 weeks	
Level of resource mismatch	{0,25,50,75,100} %<br>{0,100} %	
Mutation rate	0.01	
Transmission factor	See table A5	
Survival times	See table A5	

Table A2: Monthly reproduction probabilities (Boitani et al., 1995) used to stochastically determine the number of breeding females.

Month	Jan	Feb	Mar	Apr	May	Jun	Jul	Aug	Sep	Oct	Nov	Dec
Number of weeks	4	4	5	4	4	5	4	4	5	5	4	4
Reproduction probability	0.0	0.1	0.23	0.34	0.07	0.08	0.06	0.03	0.03	0.0	0.0	0.0

Table A3: Breed count distribution (Bieber & Ruf, 2005) used to estimate litter sizes.

Litter size	0	1	2	3	4	5	6	7	8	9	10	0
Probability	.01306	.06915	.01629	.24994	.24994	.01629	.06915	.01910	.00343	.0004	.00002	.01306

Table A4: Initial age distribution (Kramer-Schadt et al., 2009) used to initialize each model run.

Age (years)	1	2	3	4	5	6	7	8	9	10
Proportion	0.38	0.24	0.15	0.09	0.06	0.03	0.02	0.01	0.01	0.01

Table A5: Pathogen strains with their respective transmission factor and the survival time (weeks) they impose on infected hosts.

**Strain**	1	2	3	4	5	6	7	8	9	10	11	12
**Transmission factor**	.0006	.0007	.0008	.0009	.001	.002	.003	.004	.006	.007	.007	.007
**Survival time (weeks)**	14	13	11	11	10	9	8	6	3	3	2	1

References

Artois, M., Depner, K.R., Guberti, V., Hars, J., Rossi, S., Rutili, D., 2002. Classical swine fever (hog cholera) in wild boar in Europe. Rev. Sci. Tech. OIE 21, 287–303. https://doi.org/10.20506/rst.21.2.1332

Bates, D., Mächler, M., Bolker, B. and Walker, S. 2015. Fitting Linear Mixed-Effects Models Using lme4. - Journal of Statistical Software 67: 1–48.

Bieber, C., Ruf, T., 2005. Population dynamics in wild boar *Sus scrofa* : ecology, elasticity of growth rate and implications for the management of pulsed resource consumers: *Population dynamics in wild boar*. Journal of Applied Ecology 42, 1203–1213. https://doi.org/10.1111/j.1365-2664.2005.01094.x

Boitani, L., Mattei, L., Nonis, D., Corsi, F., 1994. Spatial and Activity Patterns of Wild Boars in Tuscany, Italy. Journal of Mammalogy 75, 600–612. https://doi.org/10.2307/1382507

Depner, K.R., Hinrichs, U., Bickhardt, K., Greiser-Wilke, I., Pohlenz, J., Moennig, V., Liess, B., 1997. Influence of breed-related factors on the course of classical swine fever virus infection. Veterinary Record 140, 506–507. https://doi.org/10.1136/vr.140.19.506

Depner, K.R., Müller, T., Lange, E., Staubach, C., Teuffert, J., 2000. Transient classical swine fever virus infection in wild boar piglets partially protected by maternal antibodies. Dtsch Tierarztl Wochenschr 107, 66–68.

Durio, P., Gallo Orsi, U., Macchi, E., & Perrone, A. (2014). Structure and monthly birth distribution of a wild boar population living in mountainous environment. Journal of Mountain Ecology, 3(0). Retrieved from http://www.mountainecology.org/index.php/me/article/view/112

EFSA (European Food Safety Authority). (2009). Control and eradication of Classical Swine Fever in wild boar. European Food Safety Authority Journal, 932(EFSA-Q-2007-200), 1–18. Retrieved from https://efsa.onlinelibrary.wiley.com/doi/abs/10.2903/j.efsa.2009.932

Fernández, N., Kramer-Schadt, S., Thulke, H.-H., 2006. Viability and Risk Assessment in Species Restoration: Planning Reintroductions for the Wild Boar, a Potential Disease Reservoir. E&S 11, art6. https://doi.org/10.5751/ES-01560-110106

Fernández-Llario, P., Carranza, J., Mateos-Quesada, P., 1999. Sex allocation in a polygynous mammal with large litters: the wild boar. Animal Behaviour 58, 1079–1084. https://doi.org/10.1006/anbe.1999.1234

Fletcher, R.J., 2006. Emergent properties of conspecific attraction in fragmented landscapes. The American naturalist 168, 207–219. https://doi.org/10.1086/505764

Focardi, S., Toso, S., Pecchioli, E., 1996. The population modelling of fallow deer and wild boar in a Mediterranean ecosystem. Forest Ecology and Management 88, 7–14. https://doi.org/10.1016/S0378-1127(96)03804-2

Gaillard, J. M., Vassant, J., & Klein, F. (1987). Quelques caractéristiques de la dynamique des populations de sangliers (Sus scrofa scrofa) en milieu chassé. Gibier, Faune Sauvage / ONC, Office National de La Chasse, 4, 31–47.

Grimm, V., Berger, U., Bastiansen, F., Eliassen, S., Ginot, V., Giske, J., Goss-Custard, J., Grand, T., Heinz, S.K., Huse, G., Huth, A., Jepsen, J.U., Jørgensen, C., Mooij, W.M., Müller, B., Pe'er, G., Piou, C., Railsback, S.F., Robbins, A.M., Robbins, M.M., Rossmanith, E., Rüger, N., Strand, E., Souissi, S., Stillman, R.A.,

Vabø, R., Visser, U., DeAngelis, D.L., 2006. A standard protocol for describing individual-based and agent-based models. Ecological Modelling 198, 115–126. https://doi.org/10.1016/j.ecolmodel.2006.04.023

Grimm, V., Berger, U., DeAngelis, D.L., Polhill, J.G., Giske, J., Railsback, S.F., 2010. The ODD protocol: A review and first update. Ecological Modelling 221, 2760–2768. https://doi.org/10.1016/j.ecolmodel.2010.08.019

Grimm, V., Railsback, S.F., Vincenot, C.E., Berger, U., Gallagher, C., DeAngelis, D.L., Edmonds, B., Ge, J., Giske, J., Groeneveld, J., Johnston, A.S.A., Milles, A., Nabe-Nielsen, J., Polhill, J.G., Radchuk, V., Rohwäder, M.-S., Stillman, R.A., Thiele, J.C., Ayllón, D., 2020. The ODD Protocol for Describing Agent-Based and Other Simulation Models: A Second Update to Improve Clarity, Replication, and Structural Realism. JASSS 23, 7. https://doi.org/10.18564/jasss.4259

Howells, O., Edwards-Jones, G., 1997. A feasibility study of reintroducing wild boar Sus scrofa to Scotland: Are existing woodlands large enough to support minimum viable populations. Biological Conservation 81, 77–89. https://doi.org/10.1016/S0006-3207(96)00134-6

Jędrzejewska, B., Jędrzejewski, W., Bunevich, A.N., Miłkowski, L., Krasiński, Z.A., 1997. Factors shaping population densities and increase rates of ungulates in Bialowieza Primeval Forest (Poland and Belarus) in the 19th and 20th centuries. Acta Theriologica.
Jezierski W., 1977: Longevity and mortality rate in a population of wild boar. Acta theriol., 22, 24: 337—348

Kramer-Schadt, S., Fernández, N., Eisinger, D., Grimm, V., Thulke, H.-H., 2009. Individual variations in infectiousness explain long-term disease persistence in wildlife populations. Oikos 118, 199–208. https://doi.org/10.1111/j.1600-0706.2008.16582.x

Kürschner, T., Scherer, C., Radchuk, V., Blaum, N., Kramer-Schadt, S., 2021. Movement can mediate temporal mismatches between resource availability and biological events in host–pathogen interactions. Ecol. Evol. 11, 5728–5741. https://doi.org/10.1002/ece3.7478

Leaper, R., Massei, G., Gorman, M.L., Aspinall, R., 1999. The feasibility of reintroducing Wild Boar (Sus scrofa) to Scotland. Mammal Review 29, 239–258. https://doi.org/10.1046/j.1365-2907.1999.2940239.x

Liess, B., 1987. Pathogenesis and epidemiology of hog cholera. Ann Rech Vet 18, 139–145.

Melis, C., Szafranska, P.A., Jedrzejewska, B., Barton, K., 2006. Biogeographical variation in the population density of wild boar (Sus scrofa) in western Eurasia. J Biogeography 33, 803–811. https://doi.org/10.1111/j.1365-2699.2006.01434.x

Moennig, V., Floegel-Niesmann, G., Greiser-Wilke, I., 2003. Clinical signs and epidemiology of classical swine fever: a review of new knowledge. Vet J 165, 11–20. https://doi.org/10.1016/s1090-0233(02)00112-0

Morelle, K., Podgórski, T., Prévot, C., Keuling, O., Lehaire, F., Lejeune, P., 2015. Towards understanding wild boar *S us scrofa* movement: a synthetic movement ecology approach: A review of wild boar *Sus scrofa* movement ecology. Mammal Review 45, 15–29. https://doi.org/10.1111/mam.12028

Moretti, M., 2014. Birth distribution, structure and dynamics of a hunted mountain population of Wild boars (Sus scrofa L.), Ticino, Switzerland. Journal of Mountain Ecology 3.

Pepin, K.M., Davis, A.J., Beasley, J., Boughton, R., Campbell, T., Cooper, S.M., Gaston, W., Hartley, S., Kilgo, J.C., Wisely, S.M., Wyckoff, C., VerCauteren, K.C., 2016. Contact heterogeneities in feral swine: implications for disease management and future research. Ecosphere 7. https://doi.org/10.1002/ecs2.1230

Rossi, S., Pol, F., Forot, B., Masse-provin, N., Rigaux, S., Bronner, A., Le Potier, M.-F., 2010. Preventive vaccination contributes to control classical swine fever in wild boar (*Sus scrofa* sp.). Veterinary Microbiology 142, 99–107. https://doi.org/10.1016/j.vetmic.2009.09.050

Saura, S., Martínez-Millán, J., 2000. Landscape patterns simulation with a modified random clusters method. Landscape Ecology 15, 661–678. https://doi.org/10.1023/A:1008107902848

Sodeikat, G., Pohlmeyer, K., 2003. Escape movements of family groups of wild boar *Sus scrofa* influenced by drive hunts in Lower Saxony, Germany. Wildlife Biology 9, 43–49. https://doi.org/10.2981/wlb.2003.063

Spitz, F., Janeau, G., 1990. Spatial strategies: an attempt to classify daily movements of wild boar. Acta Theriologica 35, 129–149.

Wilensky, U. (1999). NetLogo (Version 6.2). Retrieved from http://ccl.northwestern.edu/netlogo/

B Additional figures

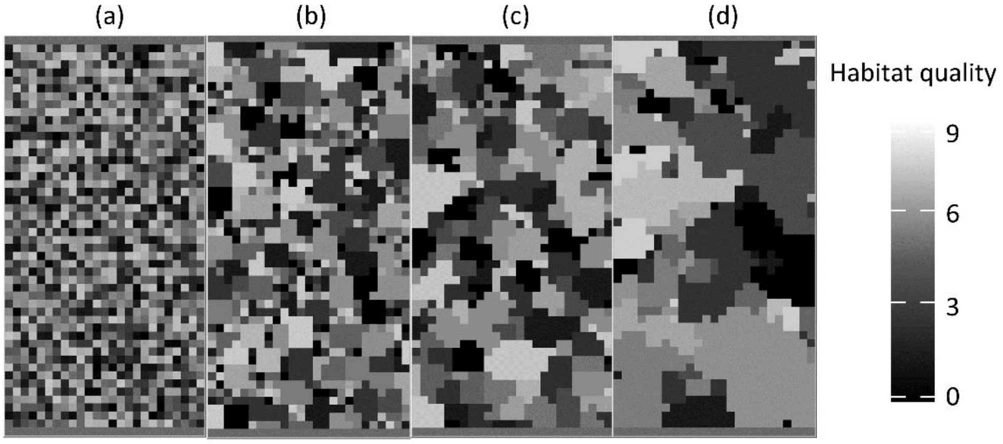

Figure B1: Example landscape configurations and habitat clustering used in the model, with (a) randomly distributed habitat cells, (b) small clusters, (c) medium clusters, (d) large clusters. The colour gradient shows the habitat quality (i.e., the number of breeding females supported by the individuals landscape cells).

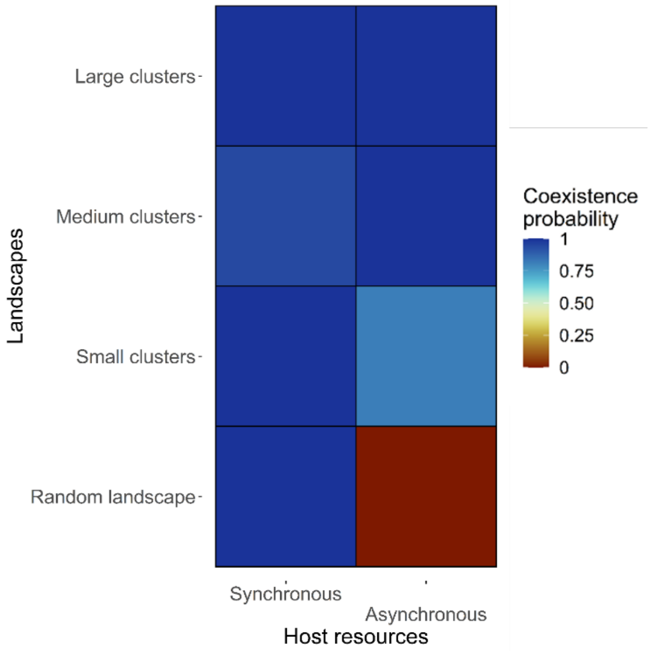

Figure B2: Coexistence probability for host and pathogen (colour gradient) for all landscape scenarios and separated for synchronous and asynchronous host resources.

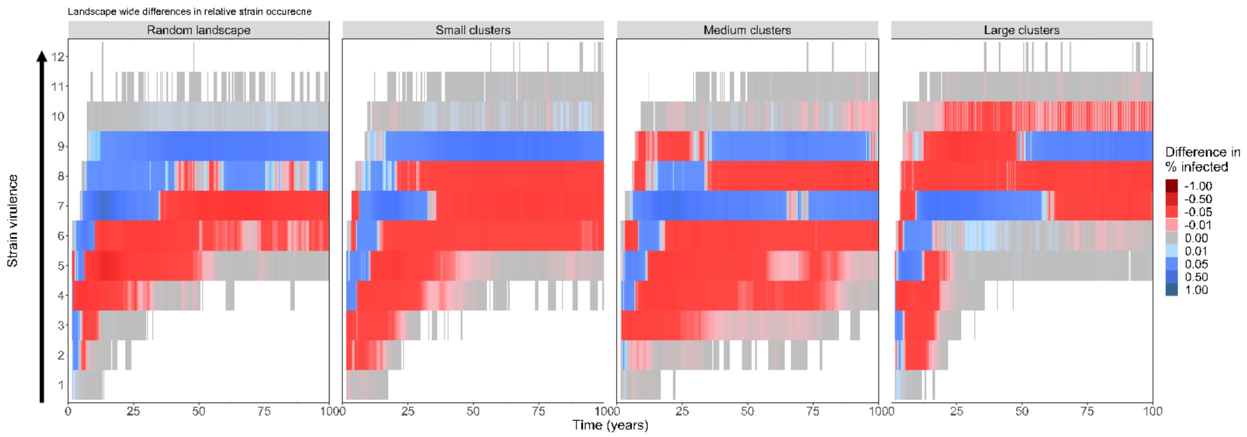

Figure B3: Differences in the proportion of infected individuals with each uncondensed strain in the landscape to highlight the effect of asynchronous scenarios. The blue colour gradient means that a strain was more dominant in synchronous (T_{lag} = 0) scenarios while the red colour gradient shows that a strain was more dominant in asynchronous scenarios (T_{lag} = 100). Gray means there was no difference in strain proportions between both scenarios.

C Generalized Linear Mixed Model – Strain prevalence

The Generalized Linear Mixed Model (GLMM) was employed to predict strain prevalence across various landscapes (panel) under both synchronous (blue) and asynchronous (red) scenarios. The analysis utilized the lme4 R package (Bates et al. 2015). The model is structured as follows:

The response variable is the strain prevalence over time (measured in weeks). This is influenced by the following explanatory variables: Landscape (i.e. configuration), Temporal lag (synchronous or asynchronous), Interaction between landscape and temporal lag and Strain.

Additionally, the model includes a random effect to account for repeated measurements.

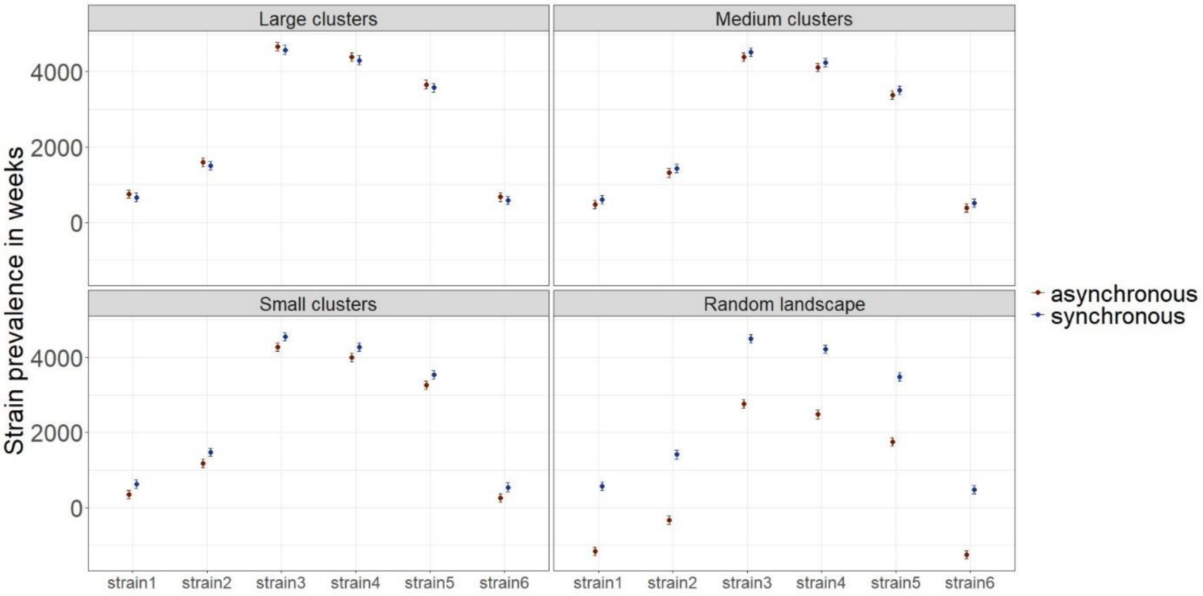

Figure C1: Glmm predicted strain prevalence for all landscapes (panel) in both synchronous (blue) and asynchronous (red) scenarios using the lme4 r package (Bates et al. 2015). Model: Strain prevalence over time ~ landscape * temporal_lag + strain + random effect (repeat).
